# Hesperidin: A Multifunctional Flavonoid with Therapeutic Potential in the Management of Pathogenesis

**DOI:** 10.3390/ijms27093806

**Published:** 2026-04-24

**Authors:** Arshad Husain Rahmani, Fahad M. Alshabrmi, Hajed Obaid A. Alharbi, Amjad Ali Khan, Fahad A. Alhumaydhi, Ahmad Almatroudi

**Affiliations:** 1 Department of Medical Laboratories, College of Applied Medical Sciences, Qassim University, Buraydah 51452, Saudi Arabia; fshbrmy@qu.edu.sa (F.M.A.); hajed.alharbi@qu.edu.sa (H.O.A.A.); f.alhumaydhi@qu.edu.sa (F.A.A.); aamtrody@qu.edu.sa (A.A.); 2 Department of Basic Health Sciences, College of Applied Medical Sciences, Qassim University, Buraydah 51452, Saudi Arabia; akhan@qu.edu.sa

**Keywords:** hesperidin, antioxidant activity, anti-inflammatory activity, health-promoting properties, pathogenesis, nanoformulation, drug synergy

## Abstract

Hesperidin, a flavonoid abundantly found in citrus fruits, has demonstrated a substantial role in the management of various pathogeneses. Furthermore, the wide range of health-promoting properties of hesperidin, including antioxidant, anti-inflammatory, anti-cancerous, hepatoprotective, neuroprotective, nephroprotective, and cardioprotective effects, has been well documented. Additionally, persuasive evidence from both in vivo and in vitro studies highlights its substantial roles in combating obesity, protecting the kidneys, liver, and lung tissue architecture, promoting wound healing, and modulating immune responses. This flavonoid acts as an effective antimicrobial agent against a wide range of microorganisms by inhibiting biofilm formation and disrupting the cell membrane. This review aims to deliver comprehensive insights into the therapeutic potential of hesperidin across different pathogenesis through distinct mechanisms. Moreover, it provides up-to-date evidence on the synergistic properties of this compound with other drugs as well as compounds, and emerging plans to enhance its efficiency in health management through various nanoformulation approaches. Despite its considerable therapeutic potential, the clinical application of hesperidin remains constrained by poor bioavailability, rapid degradation, and dosage-related limitations. Addressing these challenges will require extensive further research to clarify its mechanisms of action, safety profile, and therapeutic efficacy in managing underlying pathogenic conditions.

## 1. Introduction

The World Health Organization (WHO) states that 80% of people worldwide rely on medicinal plants for the treatment or relief of various illnesses [1]. Every day, new bioactive molecules sourced from natural resources are discovered; however, only a small number of these molecules undergo evaluation for their potential as drug candidates [2]. There has been growing attention on natural plant-based products as an important source of bioactive compounds that can contribute to therapeutic effects by influencing various biological processes. Natural products are rich sources of flavonoids, which are essential for maintaining health and managing disease. Flavonoids are a group of polyphenol compounds, including flavanols, flavones, chalcone, and others, with a C6-C3-C6 structure [3]. Flavonoids are generally present in fruits as well as vegetables and are documented as important plant-derived ingredients. Their noteworthy role in the prevention of various diseases due to their biological and pharmacological properties has been evidenced.

**Figure 1:**
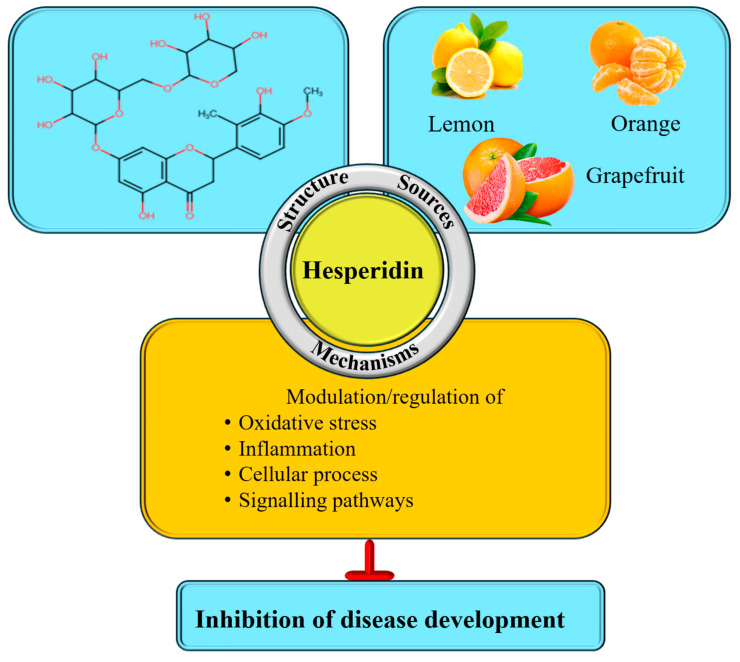
Overview of hesperidin sources, chemical structure, and mechanisms in disease prevention. The figure highlights its natural sources (primarily citrus fruits), chemical structure, and involvement of key cellular processes in disease. The chemical structure was drawn by online website (https://www.rcsb.org/search/chemical, accessed on 19 April 2026) “Draw or edit chemical structure Marvin JS by Chemaxon”.

Hesperidin is a flavonoid classified as a flavanone and is commonly found in high concentrations in citrus fruits (Figure 1). This compound has also been identified in various members of the Rutaceae family, bergamot fruits [4], bananas, lemons, and lemon peel [5]. The amount of this flavonoid found in citrus fruits varies significantly depending on the fruit type [6,7]. The level of hesperidin differs among citrus fruit juices. Sweet orange juice usually contains 200–600 mg/L, while clementines may comprise between 50 and 850 mg/L [8]. In mandarins, the concentration ranges from 8.1 to 460 mg/L, while lime as well as lemon juices contain around 38–410 mg/L [8]. Grapefruit juice generally has lower levels, ranging from 20 to 170 mg/L [8].

Hesperidin plays a substantial role in the management of various pathogeneses, mainly due to its powerful antioxidants and anti-inflammatory activities (Figure 1). Beyond its well-established anti-inflammatory and antioxidant properties, hesperidin shows a versatile range of biological activities. Previous studies demonstrate hesperidin, acting as an antioxidant, prevent oxidative stress induced by exercise as well as improve exercise performance [9]. Moreover, hesperidin demonstrates strong antioxidant and neuroprotective effects in brain tissue, protecting against diabetes-induced oxidative damage in STZ-induced rodent models [10]. It reduces oxidative stress and promotes recovery in kidney cells from oxidative injury by enhancing antioxidant activity [11], prevents oxidative stress in renal as well as liver tissues [12], and reduces tumor cancer growth [13,14,15].

This review highlights the pharmacological activities of hesperidin and studies the possible mechanisms through which it contributes to disease management. Additionally, it explores recent advancements in enhancing the bioavailability as well as the therapeutic efficacy of hesperidin, with specific emphasis on nanoformulation and nanotechnology-based delivery strategies.

## 2. Methodology

A comprehensive narrative literature review was conducted to identify studies assessing the role of hesperidin in managing various pathogeneses. The search utilized keywords such as antioxidant role, anti-inflammatory properties, and its effects on hepatoprotection, cardiovascular health, neuroprotection, as well as its involvement in cancer, obesity, and the digestive and respiratory systems Additionally, the search included terms related to the synergistic effects and nanoformulation of hesperidin. The search covered publications published between November 1987 and January 2026. A wide-ranging search was conducted across numerous electronic databases, including Web of Science, Google Scholar, PubMed, as well as Scopus. Initially, 262 articles, including review and research articles, were investigated. After excluding similar studies, a total of 233 articles were considered. Studies were included based on predefined criteria, focusing on original research and review articles published in English. The final selection includes studies representing a distribution of research types, including clinical studies, in vitro, and in vivo. Exclusions were made for non-English publications, editorials, commentaries, and conference abstracts lacking full texts.

## 3. The Pharmacological Potential of Hesperidin in Addressing Pathogenesis by Influencing Various Biological Activities

Found chiefly in citrus fruits, hesperidin has been described to affect numerous disease pathways by its multifaceted biological activity. Additionally, hesperidin’s involvement in cancer has been validated through its modulation of cell signaling pathways. It has also been shown to influence many diseases by upregulating and downregulating various activities, thereby maintaining tissue architecture. This section offers a wide-ranging overview of the role of hesperidin in several diseases, with comprehensive insights presented as follows:

### 3.1. Antioxidant Potential

Oxidative stress (OS) is defined as a disproportion between pro-oxidants as well as antioxidants that leads to dysregulation of redox processes and harm to macromolecules [16]. The imbalance between free radical formation and the body’s capability to detoxify them or repair the resultant damage leads to oxidative stress [17]. This imbalance can be a consequence of increased free radical production, reduced antioxidant defenses, or both [17]. Oxidative stress is harmful because free radicals interact with essential cellular components such as DNA, lipids, and proteins [18]. Phytochemicals are known to directly neutralize reactive oxygen species (ROS) and enhance the expression of cellular antioxidant enzymes, thereby protecting against oxidative stress-induced cellular damage [19]. Natural and bioactive compounds are vital for disease prevention because they can alleviate oxidative stress and increase antioxidant enzyme levels [20,21,22,23].

The impact of this flavonoid on the oxidant/antioxidant balance in lymphoid tissues following an intensive training regimen was evaluated. Rats underwent training for five weeks, which included two tests for exhaustion and three training sessions each week. During this time, the animals were administered an oral dose of 200 mg/kg of this compound or a vehicle control. Hesperidin was effective in preventing the rise in reactive oxygen species (ROS) production triggered. Furthermore, hesperidin has been shown to prevent the decline in the activities of antioxidant enzymes, for example, superoxide dismutase (SOD) and catalase, in the thymus and spleen following an additional exhaustion test [9]. In another study, it was reported as raised levels of oxidative stress markers were noticed in STZ-treated animals, while substantial reduction in the activity of enzymatic antioxidants and nonenzymatic antioxidants. Hesperidin treatment meaningfully lessened the changed levels of oxidative stress [10]. A study was designed to investigate whether hesperidin reverses oxidative damage in proximal tubular epithelial cells in the human kidney. It was demonstrated that hesperidin promotes recovery from oxidative injury in kidney cells through improving antioxidant and longevity pathways as well as reducing cellular senescence [11]. Administration of hesperidin at different doses reversed the levels of serum hepatic markers and lipid peroxidation markers, and restored the levels of renal and hepatic enzymatic antioxidants. This study established the protective role of hesperidin in reducing the toxic effects of iron in experimental rats [12]. In 2009, Kalpana et al. examined the antioxidant and protective effects of hesperidin. Their study demonstrated that hesperidin exhibited significant free radical scavenging effects and effectively protected RBC membranes against oxidative injury induced by H_2_O_2_ [24]. Acetaminophen (APAP) can cause hepatotoxicity and nephrotoxicity, as indicated by abnormal levels of antioxidant enzymes. It has also been shown to induce renal damage. However, the co-administration of hesperidin effectively reversed these signs of APAP toxicity. This outcome advocates that hesperidin may serve as a beneficial modulator in reducing oxidative stress and acetaminophen-induced toxicity [25]. Another study reported that benzopyrene induced testicular toxicity that was well-categorized biochemically and histologically and induced necrobiotic changes and pyknosis. Also, it increased malondialdehyde levels and decreased testicular reduced glutathione content.

Prior administration of hesperidin ameliorated all the biochemical and histological alterations caused by benzopyrene [26]. It was reported that hesperidin restored superoxide dismutase and glutathione peroxidase activities and improved total antioxidant capacity in H_2_O_2_-stimulated chondrocytes [27]. Another study examined the effectiveness of hesperidin in mitigating lipid peroxidation changes induced by ACN in rat brains. The findings indicated that pretreatment with hesperidin, when administered simultaneously with ACN, helped alleviate ACN-induced alterations in brain lipid peroxidation [28]. Hesperidin, as an antioxidant, can reduce exercise-induced oxidative stress and enhance exercise performance [9]. The antioxidant action of hesperidin is demonstrated by increasing antioxidant activity and reducing reactive oxygen species production [9,29].

A study estimated the cardioprotective effect of hesperidin against isoproterenol-induced myocardial ischemia in rats. Isoproterenol hydrochloride was used to cause myocardial ischemia. The experimental period lasted nine days. Hesperidin was given orally to rats to measure its effects. The activities of antioxidant enzymes in the cardiac tissue of both control and treated rats were evaluated. A significant decrease in these enzyme activities was detected in isoproterenol-induced ischemic rats. However, treatment with hesperidin noticeably restored the activities of these enzymes toward normal levels. These findings designate that the cardioprotective effect of hesperidin may be attributed to its antioxidant as well as anti-lipid peroxidative properties [30]. The effect of hesperidin against arsenic trioxide-induced cardiotoxicity was examined. Fifty mice were randomly divided into five groups. HES was given as 100 or 300 mg/kg/day orally, whereas arsenic trioxide (ATO) was simultaneously given through intraperitoneal injection at a dose of 7.5 mg/kg/day for one week. The results showed that hesperidin treatment meaningfully lessened oxidative stress and reduced cardiac injury markers, while increasing antioxidant levels [31]. The effect of hesperidin on nicotine toxicity was tested. Lung injury was caused by subcutaneous injection of nicotine with a dosage of 2.5 mg/kg b.w for 5 days a week. Hesperidin was given orally at a dose of 25 mg/kg b.w. Hesperidin treatment decreased the levels of all marker enzymes, and the antioxidant status was restored to near normal [32]. Male mice were used in a study to evaluate whether hesperidin ameliorates liver ischemia/reperfusion injury. Hesperidin was given orally by gavage for three days prior to surgery. To fix the optimum dose for the 3-day hesperidin treatment, a fixed-dose study was performed using doses of 100, 200, and 400 mg/kg. It was noticed that, compared with the ischemia/reperfusion (I/R) group, the I/R + hesperidin group showed significantly higher expression levels of SOD, CAT, and other antioxidant enzymes, as well as nitric oxide [33], and hesperidin increased glutathione levels in the livers of hyperlipidemic rats [34]. The hesperidin, eriocitrin, as well as eriodictyol were evaluated for their potential to prevent oxidative stress as well as systemic inflammation caused by a high-fat diet in mice. The animals were fed either a standard diet (9.5% kcal from fat), a high-fat diet (45% kcal from fat), or a high-fat diet supplemented with hesperidin, eriocitrin, or eriodictyol for a duration of four weeks. It was noticed that hesperidin reduced hs-CRP and increased serum total antioxidant potential, in that way preventing oxidative stress in rodents fed a high-fat diet [35].

### 3.2. Anti-Inflammatory Potential

**Figure 2:**
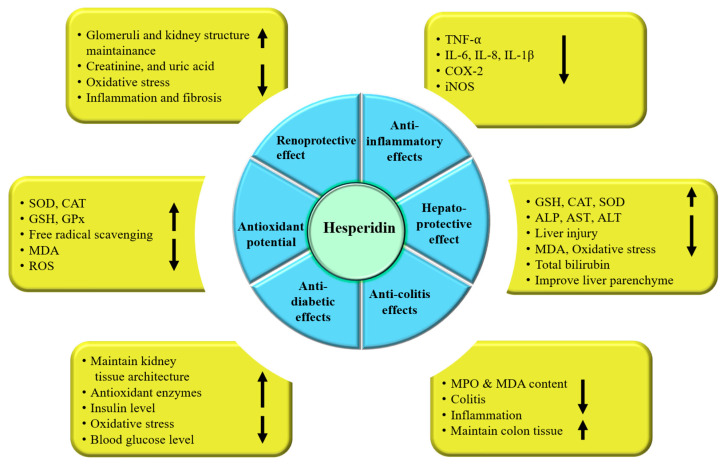
Diagram demonstrating the multifaceted pharmacological role of hesperidin in renoprotective effect, antioxidant potential, anti-diabetic potential, inhibition of digestive system diseases, hepatoprotective effect, and anti-inflammatory activity. This summarizes the associated molecular markers, enzymes, and physiological effects, with arrows indicating whether the effect is increased (↑) or decreased (↓).

Inflammation is the immune system’s reaction to harmful stimuli, including pathogens, damaged cells, toxic substances, or radiation [36]. Its primary roles are to eliminate these harmful agents and kickstart the healing process [37]. As such, inflammation serves as a crucial defense mechanism that is essential for maintaining health [38]. The detection of pathogen-linked molecular outlines and damage-related molecular patterns activates cells from the monocyte–macrophage lineage, leading to the downregulation of anti-inflammatory genes and upregulation of pro-inflammatory genes [39]. This activation results in the release of cytokines, chemokines, and other factors that induce non-specific cellular recruitment in addition to inducing vascular changes mediated via humoral factors [40]. Inflammation is often implicated in the pathogenesis of several chronic diseases [41]. It has been reported that medicinal compounds and their chief compounds act as anti-inflammatory agents [42,43,44,45,46]. Recent studies have highlighted the anti-inflammatory properties of hesperidin, demonstrating its ability to reduce levels of various inflammatory markers and enzymes, in that way suggesting its potential therapeutic role in managing inflammation-related disorders (Figure 2).

A study on pulmonary fibrosis reported that hesperidin substantially inhibited BLM-induced downregulation of lung TNF-α, IL-6, IL-1β, TGF-β, and Smad-3 mRNA expression [47]. It was reported that hesperidin improved the self-renewal ability and chondrogenesis of human mesenchymal stem cells, suppressed the expression of p65, and inhibited the secretion of pro-inflammatory cytokines [48]. It was reported that hesperidin treatment evidently decreased prostaglandin E2 and nitric oxide production and noticeably downregulated COX-2 in IL-1β-stimulated OA chondrocytes [49]. Additionally, hesperidin suppresses MAPK kinase (MEK)/ERK phosphorylation in the MAPK signaling pathway, downregulates MMP-9 expression, and decreases inflammation [50]. The anti-arthritic effects of hesperidin on the inflammatory markers of a rat model of arthritis were assessed. Female Wistar rats were used, and rats were restrained with mild inhalation of isoflurane; CFA injection was administered into the right hind paw for the arthritis induction. The study found that hesperidin might be a promising anti-inflammatory compound for the management of arthritis [51].

A study examined the role of hesperidin in mice with COPD. Cigarette smoke extract (CSE) was injected into mice to make COPD models, and they were treated with hesperidin. In the model group, mice were injected with 100% CSE (0.3 mL) at day 1, 8, and 15, whereas mice in the hesperidin-l, hesperidin-h, and budesonide groups, correspondingly, were treated with budesonide (2 mg/kg), low-dose hesperidin (25 mg/kg,) and high-dose hesperidin (50 mg/kg) daily. The levels of IL-6 as well as IL-8 in mouse BLAF of the model group presented a noticeable upward trend. After treatment, IL-6 and IL-8 levels in the mouse BLAF in the budesonide, hesperidin-l, and hesperidin-h groups were decreasing. It was reported that hesperidin, as well as budesonide, efficiently alleviated pathological changes [52]. A study intended to clarify the potential of hesperidin, which shows anti-inflammatory activity using LPS-caused inflammation mouse model. LPS was administered by injection. The Hes plus LPS group was fed AIN-93G comprising 0.1% hesperidin and subjected to LPS injection. Dietary hesperidin was found to reduce systemic LPS-induced signs of illness, including elevated blood leukocyte counts [53]. A study was planned to estimate the ameliorative role of hesperidin (HSP) on toxicity in the heart of rats. NaF (600 ppm, orally) was added to the water of the rats and given for 14 days. NaF plus HSP (100 and 200 mg/kg, b.w.) was given. Hesperidin treatment reduced the sodium fluoride (NaF)-induced heart tissue injury. It changes the expression of apoptotic markers and the levels of autophagic and inflammatory parameters in NaF-induced cardiotoxicity [54].

A study confirmed that hesperidin suggestively attenuated HFFD-induced oxidative stress as well as inflammation by dropping plasma malondialdehyde levels and aortic superoxide anion production, as well as by suppressing the overexpression of aortic TNF-α as well as IL-6 [55]. It was proposed that prolonged hesperidin administration influences the inflammatory response and oxidative stress levels in animals subjected to mechanical ventilation [56]. The hesperidin effect against colon toxicity was noted, as hesperidin decreased the expression of inflammatory and apoptotic markers [57]. A study assessed the chemopreventive potential of hesperidin against hepatocarcinogenesis induced by diethylnitrosamine (DEN). Rats were administered a single IP injection of DEN (200 mg/kg), followed two weeks later by subcutaneous injections of CCl_4_ (3 mL/kg, once weekly) for 16 weeks, and 0.5% CMC through oral gavage daily for 18 weeks. The anti-inflammatory effects of hesperidin (50 and 100 mg/kg) were assessed by measuring serum TNF-α levels. DEN/CCl_4_ exposure meaningfully raised circulating TNF-α, whereas treatment with hesperidin at both doses distinctly reduced its levels. As well, hesperidin significantly suppressed NF-κB expression in the liver. DEN/CCl_4_-induced rats also showed increased hepatic TGF-β1 expression and Smad3 phosphorylation; these alterations were attenuated by hesperidin treatment at either 50 or 100 mg. Overall, hesperidin improved liver function by lowering circulating hepatic enzyme markers as well as TNF-α [58].

### 3.3. Anti-Diabetic Effects

Diabetes is characterized by metabolic imbalances affecting fats, proteins, and carbohydrates. It occurs due to insufficient insulin production, high blood sugar levels, or a combination of both, leading to various metabolic alterations that can lead to death [59,60,61]. The current methods for treating diabetes can lead to adverse effects. However, research has shown that natural products have anti-diabetic potential through various mechanisms [62,63]. In this context, hesperidin’s role as an anti-diabetic agent is designated (Table 1 and Figure 2).

A study was designed to examine the potential effect of hesperidin against type 2 diabetes mellitus (T2DM). Male Sprague–Dawley rats were orally administered 100 mg/kg hesperidin or vehicle for 35 days. It was demonstrated that fasting serum glucose levels improved, while insulin levels remained unchanged by the treatment of hesperidin. This indicates that hesperidin might aid in averting the development of insulin resistance and diabetes by enhancing insulin sensitivity [64]. The glucose uptake assay showed that hesperidin treatment restored glucose uptake to normal levels, independent of insulin. In HFD-induced obese mice, administration of hesperidin led to decreases in blood glucose, as well as serum insulin. Moreover, hesperidin treatment improved insulin and glucose tolerance to normal levels [65]. The effects of hesperidin’s antioxidant and hypoglycemic properties on diabetic nephropathy were studied. In the diabetic group, there were raised levels of serum urea, malondialdehyde, and creatinine. In contrast, hesperidin therapy pointedly controlled these values in diabetic rats [66]. Another study found that hesperidin, as well as quercetin, enhanced the activities of CAT, SIRT1, and SOD in kidney tissues of the DM+HP groups compared with the DM group [67]. The rats were given streptozotocin (STZ, 45 mg/kg), a single dose intraperitoneally, to cause diabetic neuropathic pain. The HES groups were treated with 100 mg/kg and intragastric gavage daily. The outcomes exhibited that HES treatment in diabetic rats reduced STZ-induced hyperglycemia as well as thermal hyperalgesia. Additionally, in the histopathological experimentation of the sciatic nerve, HES treatment reduced STZ-induced injury [68]. Satoko Akiyama et al. (2009) reported that dietary hesperidin intake normalized lipid and adiponectin levels and reduced blood glucose [69]. Additionally, higher serum insulin levels were noted in the hesperidin group. A study investigated the effect of hesperidin on enzymes involved in carbohydrate metabolism in STZ-induced diabetic rats. The diabetic condition was induced by streptozotocin (40 mg/kg body weight), after which hesperidin was given at doses of 25, 50, and 100 mg/kg b.w for a duration of 30 days. It was exhibited that in the hesperidin-treated group, plasma glucose levels dramatically decreased in a dose-dependent way, highlighting the effects of this compound [70]. Male rats were made diabetic using intraperitoneal injection of STZ (65 mg/kg), and then 100 mg/kg hesperidin was administered daily over 4 weeks. Administration of hesperidin to diabetic rats resulted in reduced fasting blood glucose levels and elevated serum and pancreatic insulin levels [71]. A study was conducted to examine the anti-diabetic effect of hesperidin on streptozotocin (STZ)-induced diabetes mellitus in mice. The hesperidin with doses of 100, 200, and 400 mg/kg was given. All groups received treatment once daily for 21 successive days, excluding the normal and diabetic control groups. The diabetic control mice showed a marked increase in glucose levels, and the animals treated with insulin or hesperidin demonstrated a significant decrease in glucose levels [72]. Hesperidin supplementation ameliorated and improved raised glycosylated hemoglobin and glucose levels while also enhancing serum insulin levels in insulin-resistant diabetic animals [73]. A study aimed to explore the role of hesperidin and naringin in oxidative damage caused by hyperglycemia in rats with HFD/STZ-induced diabetes. Diabetes was induced by feeding the rats a HFD, followed by an intraperitoneal injection of STZ at a dose of 35 mg/kg body weight. After diabetes was induced, the rats received a daily oral dose of 50 mg/kg of either hesperidin or naringin for a duration of four weeks. In untreated diabetic rats, blood glucose and glycosylated hemoglobin levels were increased, whereas serum insulin levels were reduced. Treatment with hesperidin or naringin appeared to evidently counteract these alterations [74]. A study was designed to estimate the antihyperlipidemic and antihyperglycemic roles of a *Citrus reticulata* fruit peel hydroethanolic extract, along with hesperidin as well as quercetin, in nicotinamide/STZ-induced diabetic rats. The findings concluded that the extract, quercetin, and hesperidin show remarkable anti-diabetic activity, which may be attributed to their insulinotropic properties and insulin-sensitizing activities [75].

**Table 1:** Anti-diabetic property of hesperidin through different mechanisms.

Study Model	Doses	Animal Species	Treatment Duration	Key Findings	Ref.
Alloxan and HFD-induced insulin resistance model	100 mg/kg	Male Sprague–Dawley rats	35 days	° Improved fasting serum glucose levels ° Regulated both glycolysis and gluconeogenesis	[64]
STZ-induced diabetic neuropathy model	100 mg/kg	Male Sprague–Dawley rats	14 days	° Decreased hyperglycemia and thermal hyperalgesia ° Reduced neuronal damage and improved neuropathy	[68]
Streptozotocin-induced marginal diabetic model	10 g/kg diet	Male Wistar rats	4 weeks	° Lowered blood glucose levels and increased serum insulin concentration	[69]
Streptozotocin-induced diabetic model	25, 50, 100 mg/kg	Male albino Wistar rats	30 days	° Decrease in plasma glucose levels noticed	[70]
Streptozotocin-induced diabetic model	100 mg/kg	Male Sprague–Dawley rats	Over 4 weeks	° Protects pancreatic β-cells and enhanced their function	[71]
Streptozotocin-induced diabetic model	100, 200, 400 mg/kg	Swiss Albino mice	21 days	° Decreased glucose, ALP, and AST levels	[72]
High-fat diet/STZ-induced diabetic model	50 mg/kg	Male albino rats	30 days	° Antihyperglycemic and anti-dyslipidemic effects noticed ° Improved cardiac function	[73]
High-fat diet/STZ-induced diabetic model	50 mg/kg	Male albino rats	4 weeks	° Decreased glucose levels and increased serum insulin levels	[74]
Nicotinamide/STZ-induced diabetic model	100 mg/kg	Male Wistar rats	4 weeks	° Caused antihyperlipidemic and antihyperglycemic effects	[75]

### 3.4. Hepatoprotective Potential

Liver disease remains a main global health issue, meaningfully affecting mortality rates globally. Natural products as well as their bioactive ingredients have shown auspicious hepatoprotective effects. These substances can help lessen liver damage by lowering oxidative stress and inflammation, regulating the activity of liver enzymes, as well as maintaining the structural integrity of liver tissue [76,77,78,79]. The hepatoprotective properties of hesperidin have been demonstrated through multiple mechanisms (Table 2 and Figure 2).

The role of hesperidin (100 or 200 mg/kg) as an anti-fibrotic potential against liver fibrosis in rats was investigated. Dimethylnitrosamine was administered to cause liver fibrosis in rats. Dimethylnitrosamine caused liver fibrosis, as evidenced by higher levels of liver function enzymes and both total as well as direct bilirubin. Additionally, a decline was detected in serum total protein, hepatic glutathione levels, and albumin. However, treatment with hesperidin efficiently mitigated the harmful effects of dimethylnitrosamine across all measured parameters [80]. Cetin et al. reported that CCl4 exposure caused liver oxidative stress and decreased activities of antioxidant enzymes. CCl4 also increased caspase-3 levels, leading to hepatocyte apoptosis. Notably, hesperidin (orally administered at 50 mg/kg/day) counteracted these changes, with its mechanism likely involving antioxidant and anti-apoptotic properties. Hesperidin treatment mitigated the effects of CCl4 and promoted anti-apoptotic processes, as shown by a decline in caspase-3 activation [81]. A noteworthy impairment in liver function was detected, indicated by an increase in serum levels of liver enzymes and total bilirubin. There were increased levels of serum and tissue nitrite. Moreover, the administration of LPS led to elevated tTBARS levels, while antioxidant enzyme activity in the liver homogenates of rats showed a reduction, highlighting noteworthy oxidative stress. Treatment with hesperidin effectively mitigated these LPS-induced effects [82].

The protective role of HDN in concanavalin A (Con A)-induced hepatic injury was checked. An acute hepatic injury model was made by intravenous administration of Con A in mice, and HDN was given for 10 days before Con A challenge. The protective effects of hesperidin on Con A-induced hepatic damage were evaluated. It was observed that the hepatic damage was remarkably improved in hesperidin-pretreated mice [83].

A study investigated the protective effects of hesperidin and ellagic acid against hepatotoxicity. Rats were administered a sub-lethal oral dose of mercuric chloride to induce toxicity. Mercuric chloride treatment in rats caused a substantial decrease in the activities of antioxidant enzymes and an increase in free radical levels in the liver. When rats were also treated with hesperidin (5 mg/kg b.w) alongside mercuric chloride, these negative changes were improved. Overall, this study confirmed the hepatoprotective effects of hesperidin as well as ellagic acid [84].

**Table 2:** Hepatoprotective property of hesperidin through various mechanisms, such as reduction of liver function enzymes, oxidative stress, inflammation, and maintenance of hepato-tissue structure.

Study Model	Doses	Animal Species	Treatment Duration	Key Findings	Ref.
Dimethylnitrosamine-induced liver fibrosis model	100 or 200 mg/kg	Male Wistar rats	4 weeks	° Decline liver function enzymes ° Increase hepatic glutathione content	[80]
Carbon tetrachloride-induced hepatotoxicity model	50 mg/kg	Wistar albino rats	10 days	° Blocked the toxic effects and increased antioxidant enzymes levels	[81]
Lipopolysaccharide-induced hepatotoxicity model	50, 100, 200 mg/kg	Male Wistar rats	7 days	° The levels of liver function enzymes decreased	[82]
Arsenite-induced hepatotoxicity model	100, 200 mg/kg	Male Sprague–Dawley rats	15 days	° Reduced apoptosis, oxidative stress, and inflammation	[85]
Bile duct ligation-induced liver injury model	100, 200 mg/kg	Male Wistar rats	21 days	° Hesperidin supplementation decreased liver enzymes ° Decreased bilirubin and malondialdehyde	[86]
Paclitaxel-induced hepatotoxicity model	10 mg/kg	Male Wistar rats	6 weeks	° The hesperidin treatment reduced liver function enzymes and liver lipid peroxidation level	[87]
Diclofenac-induced hepatotoxicity model	20 mg/kg	Male Wistar rats	4 weeks	° Protects liver injury	[88]

A study aimed to evaluate the role of hesperidin (HSP) against kidney and liver damage caused by sodium arsenite (SA) in rats. The animals were administered SA (10 mg/kg b.w) along with HSP at doses of 100 and 200 mg/kg body weight through oral gavage for a duration of 15 days. HSP administration meaningfully lowered inflammation, apoptosis, and oxidative stress in liver tissues affected by sodium arsenite, with effects varying by dosage. This study indicated that HSP provides a hepatoprotective effect [85].

A study was conducted to estimate the hepatoprotective role of hesperidin in BDL-induced liver injury in rats. Treatments were orally provided for 21 consecutive days. Hesperidin supplementation was related to a noteworthy reduction in MDA levels, liver enzymes, inflammatory gene expression, bilirubin, and nitric oxide. Furthermore, it led to substantial increases in glutathione, superoxide dismutase, total antioxidant capacity, and catalase activity. Furthermore, there was a marked improvement in the histological morphology as well as structure of the liver parenchyma [86].

The hesperidin as well as rutin administration to paclitaxel-treated rats meaningfully mitigated the elevations in serum aspartate transaminase, alanine transaminase, gamma-glutamyl transferase, and alkaline phosphatase, along with liver lipid peroxidation and total bilirubin levels. Furthermore, the adverse hepatic histological changes induced by paclitaxel were markedly improved with hesperidin and rutin treatment [87]. A study intended to assess the protective effects of naringin, hesperidin, and their combination, as well as to explore their possible mechanisms of action in diclofenac-induced liver toxicity. Rats received intraperitoneal injections of diclofenac sodium (3 mg/kg b.w) and were orally administered naringin (20 mg/kg b.w) and hesperidin (20 mg/kg b.w) for 4 weeks. Treatment with either hesperidin or naringin in diclofenac-exposed rats meaningfully lowered the enhanced serum levels of AST, ALT, ALP, GGT, IL-17, as well as TNF-α. Moreover, histopathological damage induced by diclofenac in the liver was significantly ameliorated following treatment with hesperidin or naringin [88]. The role of rutin as well as hesperidin in experimental doxorubicin-induced hepatotoxicity was checked. Doxorubicin (DXR)-administered rats (25 mg/kg; intraperitoneally for two weeks) were pretreated with rutin, hesperidin, or their mixture (50 mg/kg b.w) three times per week for three weeks. The investigation found that administering doxorubicin to rats caused a noteworthy increase in serum levels of liver enzymes. There was also a prominent decrease in antioxidant enzymes. These findings advocate that HSP and rutin alleviate doxorubicin-induced liver toxicity by restoring the changed parameters [89].

### 3.5. Renoprotective Effects

Medicinal plants and their ingredients exhibit noteworthy renoprotective effects. Among them, hesperidin has been shown in studies to confer renal protection through various mechanisms (Table 3, Figure 2).

A study inspected the effects of chronic exposure to dusty particulate matter (PM) and evaluated the role of hesperidin (HSP) in the renal tissue in rats. In the dust storm (DS) groups, the rats were exposed to dust concentrations of 5000–8000 μg/m^3^ in a chamber for one hour daily for four weeks. In a real-like dust storm (DS) model, blood urea nitrogen and creatinine, an inflammatory marker, and oxidative stress indices were enhanced. Similarly, a significant increase was observed in collagen-I, mRNA, and Smad expression, as well as in fibrosis in the kidney. However, hesperidin meaningfully reverses such changes [90].

Another study reported that the ischemia–reperfusion (I/R) group showed lower expression of antioxidant enzymes and higher expression of serum creatinine as well as blood urea nitrogen. In comparison to the I/R group, the I/R-hesperidin group exhibited increased levels of antioxidant enzymes and decreased levels of serum creatinine and blood urea nitrogen. Hesperidin facilitated the improvement of renal I/R injury through its antioxidative properties [91]. The renoprotective effect of hesperidin against gentamicin (GEN)-caused nephrotoxicity was checked. Gentamicin (GEN) was injected intraperitoneally (i.p.) at a dose of 100 mg/kg B.W to induce nephrotoxicity. HDN (200 mg/Kg b.w) was orally given to evaluate the effects. Renal damage was shown by increased serum urea, creatinine, as well as uric acid. It diminished antioxidant enzyme levels and glomerular atrophy and necrosis in proximal tubules. Supplementation with hesperidin in gentamicin-induced rats reduced uric acid, urea, creatinine, TBARS, and NO generation, and increased antioxidant enzyme content compared with gentamicin alone [92]. The renoprotective potential of hesperidin as well as sitagliptin was examined in an experimental model. Nephrotoxicity was induced using cyclosporine A (CsA) at a dose of 20 mg/kg/day administered i.p for seven days. Treatment groups received sitagliptin (10 mg/kg/day) or hesperidin (200 mg/kg/day) orally for 14 days. Both sitagliptin as well as hesperidin reduced CsA-caused increases in serum creatinine, blood urea, renal MDA, glucose, and MPO, while maintaining normal levels of serum albumin and enhancing renal antioxidant markers [93].

A study was designed to explore the protective effect of hesperidin against renal damage induced by AlCl_3_. The rats were allocated into four groups: a control group, a hesperidin-treated group (80 mg/kg b.w, orally), an AlCl_3_-treated group (10 mg/kg body weight), and a combined treatment group receiving both AlCl_3_ and hesperidin. ALCL3 induced renal damage in rats, showing renal histopathological damage and an intense decline in renal function. Hesperidin meaningfully reversed all the above-mentioned damaging effects [94]. The effect of hesperidin (HES) and rutin (RUT) against cisplatin-induced nephrotoxicity in rats was examined. The administration of HES (200 mg/kg, per oral) or RUT (30 mg/kg, p.o.) for 14 days with one cisplatin dose (5 mg/kg, ip) on the tenth day ameliorated the cisplatin-induced nephrotoxicity, as designated by the restoration of oxidative stress biomarkers and kidney function. Additionally, it reduced the histopathological deviations caused by cisplatin [95].

Hesperidin pretreatment improved the histopathological as well as biochemical indicators in the kidney tissues of rats with ischemia–reperfusion injury [96]. A study assessed the renoprotective role of hesperidin as well as eugenol against cadmium-induced kidney toxicity. Treatment with hesperidin (100 mg/kg b.w daily) and eugenol led to reduced serum urea and creatinine levels, along with improved renal tissue structure, when compared to the cadmium-treated group [97].

**Table 3:** Renoprotective property of hesperidin through various mechanisms, such as antioxidant, anti-inflammatory, and reduction of renal injury.

Study Model	Doses	Animal Species	Treatment Duration	Key Findings	Ref.
Kidney injury induced by dust storm particulate matter model	200 mg/kg	Male Wistar rats	28 days	° Hesperidin decreased blood urea nitrogen and creatinine ° This compound inhibited histological changes and decreased renal fibrosis	[90]
Ischemia-induced kidney injury model	100 mg/kg	Sprague–Dawley rats	7 days	° Improved renal injury by its antioxidant effects	[91]
Cyclosporine-induced kidney damage model	200 mg/kg	Male Wistar rats	14 days	° Hesperidin protects kidney	[93]
Aluminum-induced renal injury model	80 mg/kg	Male Wistar rats	5 weeks	° It decreased apoptosis inflammation ° Reduced renal histopathological damage	[94]
Cisplatin-induced nephrotoxicity model	200 mg/kg	Male Sprague–Dawley rats	14 days	° Protective effects against nephrotoxicity	[95]
Cadmium-induced nephrotoxicity model	200 mg/kg	Male Wistar rats	4 weeks	° Protected from the functional and structural kidney injury induced through antioxidant and anti-apoptotic effects.	[97]

### 3.6. Cardioprotective Effects

Natural compounds have proven cardioprotective potential. In this, the cardioprotective property of hesperidin through various mechanisms has been described by different findings (Table 4). Cardiovascular diseases represent a serious challenge for healthcare systems worldwide. The cardiotoxicity of DOX and the cardioprotective role of HES were checked. Groups of rats were either treated with DOX (4 mg/kg. bw., ip) or received co-treatment with HES (100 mg/kg. bw./day orally, for five consecutive weeks). The administration of doxorubicin (DOX) led to noteworthy cardiotoxic effects, as shown by marked increases in cardiac biomarkers, for instance, creatine kinase isoenzyme MB, Troponin I, creatine, and lactate dehydrogenase. DOX also resulted in a disarray of cardiomyocytes, infiltration of inflammatory cells, and congestion of cardiomyocytes. However, co-treatment with hesperidin provided protection to cardiac tissues against the cardiotoxic effects induced by DOX [98]. A study explored the cardioprotective effects of hesperidin against CO-induced cardiac damage. To establish CO toxicity, rats were exposed to CO, and hesperidin (25, 50, and 100 mg/kg/day) was given intraperitoneally. Hesperidin treatment was found to decrease myocardial necrosis. At a dose of 100 mg/kg, it also enhanced Akt protein expression and lowered the BAX/BCL2 ratio. Overall, hesperidin attenuated the detrimental cardiac effects caused by carbon monoxide poisoning [99].

A study evaluated the protective role of hesperidin (HES) against cisplatin (CP)-induced cardiotoxicity. Mice were given HES orally at doses of 100 or 300 mg/kg/day for 7 consecutive days, while cisplatin (5 mg/kg) was given intraperitoneally on days 3 and 6. Treatment by HES lessened CP-induced cardiac tissue damage and decreased the leakage of myocardial injury markers. Additionally, HES suppressed the release of inflammatory mediators triggered by cisplatin [100].

**Table 4:** Cardioprotective property of hesperidin such as reduction of cardiac necrosis and heart damage.

Study Model	Doses	Animal Species	Treatment Duration	Key Findings	Ref.
Doxorubicin-induced cardiotoxicity model	100 mg/kg	Male Wistar albino rats	5 weeks	° Hesperidin proves cardioprotective effects	[98]
CO-induced cardiac injury model	25, 50, and 100 mg/kg	male Wistar rats	5 days	° Decreased cardiac necrosis	[99]
5-Fluorouracil-induced cardiotoxicity model	100 mg/kg	Male Wistar albino rats	8 days	° Hesperidin and TQ treatment lowered cardiac enzymes and diminished inflammation	[101]
Streptozotocin-induced cardiac damage	100 mg/kg	Wistar Albino rats	14 days	° Hesperidin exhibited protective effects through the modulation of inflammation, oxidative stress, and apoptosis.	[102]
Paclitaxel-Induced Nephrocardiotoxicity model	10 mg/kg	Male Wistar rats	6 weeks	° The cardiac dysfunction reduced ° Severity of the heart’s histopathological decreased	[103]
Arsenic trioxide-induced cardiac toxicity model	100 mg/kg	Male albino Wistar rats	30 Days	° Ease cardiac toxicity and histopathological damage	[104]
Doxorubicin-induced cardiomyopathy model	50 mg/kg	Males Sprague–Dawley rats	3 weeks	° Hesperidin improved cardioprotective effects	[105]
Hypertensive animal model	30 mg/kg	Wistar–Kyoto rats	25 weeks	° Hesperidin showed anti-hypertensive effects	[106]

A study examined the efficacy of hesperidin (100 mg/kg orally for eight days) and thymoquinone (TQ) in preventing cardiotoxicity. Treatment with TQ as well as HESP lowered cardiac enzyme levels and diminished inflammation in cardiac tissues [101].

A study examined the cardioprotective effects of hesperidin (HES) against cardiac damage induced by STZ-mediated diabetes in rats. Diabetes was caused by STZ (45 mg/kg), and HES (100 mg/kg/day) were administered intragastrically to treatment groups for 14 days. STZ administration meaningfully increased TNF-α expression, MDA, and TOS levels, and reduced GSH and TAS levels, accompanied by severe myocardial degeneration and vascular and congestion necrosis. HES treatment evidently improved antioxidant status, decreased oxidative and inflammatory markers, and reduced apoptosis. Histopathological assessments demonstrated considerable preservation of cardiac morphology [102]. Hesperidin treatment meaningfully reduced cardiac dysfunction in rats treated with paclitaxel. After the administration of paclitaxel, the heart’s histopathological findings were notably reduced due to hesperidin treatment [103]. The cardioprotective effect of hesperidin against arsenic trioxide-caused cardiac toxicity in rats has been reported. Cardiac toxicity was induced by the administration of 4 mg/kg arsenic trioxide orally for 30 days. Hesperidin (100 mg/kg) reduced the end-diastolic velocity and peak systolic velocity, while increasing heart rate. Arsenic trioxide-caused histopathological damage to the cardiac tissue was prominently lessened by treatment with hesperidin [104]. Hesperidin (HSP) treatment reduced heart tissue damage by increasing activities of antioxidant enzymes and suppressing lipid peroxidation [54].

### 3.7. Neuroprotective Potential

Hesperidin plays an imperative role in preventing the development of nervous system pathogenesis through multiple mechanisms (Table 5). A study investigated the neuroprotective role of hesperidin (Hsd) against the changes caused by methotrexate (MTX). The rats were orally given Hsd at a dosage of 100 mg/kg for 21 days. Hesperidin prevents the neurotoxic effects of MTX via enhancing hippocampal neurogenesis and reducing oxidative stress [107]. Another study exhibited that an 8-week exposure to fluoride resulted in motor impairment, as detected by reduced fall time in the rotarod test, memory impairment, and depression-like behavior. Treatment with hesperidin (200 mg kg^−1^ d^−1^; per os) improved neurobehavioral impairment and restored brain biochemical changes [108]. Mild traumatic brain injury induction induced depressive-like behaviors by increasing oxidative stress markers and inflammatory cytokines and dropping BDNF levels in the hippocampus. Remarkably, reduced depression was induced by hesperidin treatment [109]. It was reported that hesperidin (50 mg/kg) treatment was powerful in preventing memory impairment and depressive-like behavior [110]. Hesperidin combined with L-DOPA shows anti-Parkinson properties in an animal model treated with 6-OHDA [111]. The neuroprotective effect of hesperidin against MPTP-induced behavioral deficits, inflammation, and oxidative stress was determined. It was noted that hesperidin ameliorates MPTP-induced motor dysfunction and protects against microglia activation [112]. Both dapagliflozin and hesperidin were effective in restoring behavioral test results to baseline levels. Additionally, each agent demonstrated the capacity to counteract LPS-induced alterations, as detected by electron microscopy and histopathological analysis of brain tissues [113]. A study investigated the neuroprotective activity of hesperidin against cognitive dysfunction caused by aluminum chloride (AlCl_3_). Rats received intraperitoneal injections of AlCl_3_ (100 mg/kg b.w) for 60 days, which led to impairments in learning and memory. This treatment also increased Bax expression while declining reduced glutathione levels, antioxidant enzyme activities, and Bcl-2 expression in the cerebellum, hippocampus, as well as cortex. Co-administration of hesperidin (100 mg/kg, b.w. oral) prevented the biochemical anomalies, cognitive deficits, as well as apoptosis induced by AlCl_3_ treatment [114]. Treatment with Hes (50 and 100 mg/kg) improved neurobehavioral alterations by enhancing memory retention and increasing fall-off time. Additionally, hesperidin significantly lessened histopathological changes [115]. Co-treatment with hesperidin (100 and 200 mg/kg) suggestively countered the biochemical and memory impairments caused by scopolamine administration [116].

### 3.8. Anti-Cancer Potential

**Figure 3:**
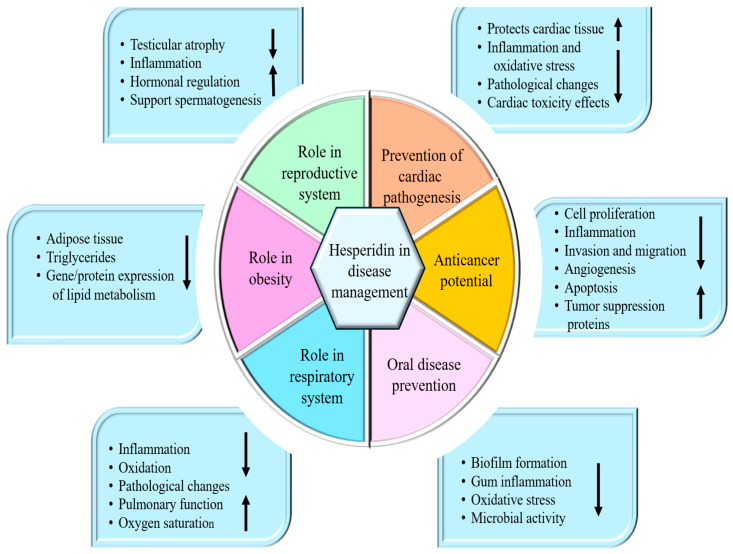
Role of hesperidin in the management of various pathogeneses. The downregulation is indicated by the downward-directing arrow, while the upregulation is denoted by the upward arrow.

Hesperidin has confirmed potential in cancer management (Table 6) by inhibiting tumor initiation, progression, invasion, and metastasis. It modulates multiple cell signaling pathways, including the suppression of angiogenesis, cell proliferation, and inflammation, while activating tumor suppressor genes, promoting apoptosis, and inducing cell cycle arrest (Figure 3). A study was performed to evaluate the role of hesperidin in glycolysis in colorectal cancer (CRC) cell lines. In vitro, SW620 and HCT116 were treated with doses of hesperidin (0–500 µmol/L), and cell counting kit-8 as well as colony formation assays were used to observe the inhibition role of hesperidin in CRC cell lines. Wound healing and transwell assays were made to observe the ability of hesperidin (0, 25, 50, and 75 µmol/L) to migrate CRC cells. The study findings revealed that hesperidin inhibits CRC cell line growth and showed an inhibitory effect on the migrating abilities of CRC cells. It significantly suppressed tumor xenograft growth in nude mice [117]. Another study based on oral carcinoma reported that a dose-dependent cytotoxic effect of hesperidin was revealed, with a notable IC50 value suggestive of its powerful cell proliferation inhibition. Treatment with this compound caused a remarkable decrease in inflammatory markers and Bcl2 mRNA expression levels, demonstrating a role in cell proliferation, migration, as well as inhibition of inflammation processes [118].

The anti-cancer role of hesperidin against prostate cancer cells was determined. The in vitro treatment of prostate cancer cells resulted in a marked reduction in cell growth and viability, with effects that were dependent on the dosage used [119]. The role of hesperidin in lung cancer through in Vitro and in vivo analyses was examined. It was noticed that pinX1 expression level is closely associated with overall survival and plays a significant part in regulating lung cancer cell proliferation, invasion, migration, and senescence. Hesperidin suggestively reduced tumor volume and tumor weight [120]. Another study based on breast cancer reported that mice treated with a combination of HES as well as doxorubicin (Dox) displayed the highest survival rate at 80%. In comparison, the survival rates of the other groups were as follows: normal saline (43%), hesperidin at doses of 5 mg/kg (54%), 10 mg/kg (55.5%), 20 mg/kg (60.5%), and 40 mg/kg (66%), and the Dox-treated group at 10 mg/kg (73%). Furthermore, there was a notable increase in the pCR score in the combination treatment group. There were significant reductions in Ki-67 as well as VEGF levels, along with a notable increase in E-cadherin [121]. The role of hesperidin in colon carcinogenesis was evaluated. Azoxymethane (AOM, administered by intraperitoneal injections (i.p.) (15 mg/kg body weight)) was given to induce a mouse model of colon carcinogenesis. Hesperidin (25 mg/kg body weight) treatment was provided either in initiation/post-initiation mode correspondingly. Hesperidin supplementation-initiated apoptosis through targeted inhibition of constitutively activated Aurora-A mediated PI3K/Akt/GSK-3β as well as mTOR pathways together with autophagic stimulation [122].

The role of hesperidin combined with carboplatin in non-small-cell lung cancer was also explored. NCI-H460 and A549 cells were treated with concentrations of hesperidin (10, 50, and 100 μM). Hesperidin inhibited the activity as well as the invasion of cells in a dose-dependent way and induced apoptosis. The combination of hesperidin and carboplatin exhibited the most noticeable anti-tumor effect [123]. Hesperidin promotes apoptosis in MCF-7 breast cancer cells by decreasing Bcl-2 protein and increasing Bax expression levels. Moreover, it also induces apoptosis in the MDA-MB-231 [124]. Another study’s result concluded that the anti-cancer effect of hesperidin in human lung cancer A549 cells was noted by p53 upregulation, which causes the proliferation arrest as well as mitochondrial-dependent apoptosis activation [125].

### 3.9. Anti-Arthritis Potential

Hesperidin role as anti-arthritis has confirmed through different mechanisms (Table 6). Another study found that treatment with hesperidin at a dose of 160 mg/kg b.w. led to noteworthy improvements in all measured parameters in rats with collagen-induced arthritis. These findings indicate that oral administration of hesperidin could be beneficial for treating rheumatoid arthritis in humans [126]. Hesperidin treatment markedly decreased prostaglandin E2 and nitric oxide production and markedly downregulated cyclooxygenase-2 expression and inducible nitric oxide synthase in IL-1β-stimulated osteoarthritis chondrocytes [49]. Collagen type II suspension was administered to rats to induce a model of rheumatoid arthritis. At the same time, hesperidin was given orally at a dose of 50 mg/kg b.w for 21 days. X-ray analysis of the rats’ hind paws designated that hesperidin treatment was remarkably effective in reducing bone loss. Furthermore, elevated levels of CML in plasma and IgG were meaningfully reduced following administration of hesperidin. Similarly, the levels of PTD in plasma and IgG were reduced to 28.46 ± 1.20 ng/mL and 359.35 ± 31.11 ng/g IgG, respectively, in hesperidin-treated rats [127]. Bee venom and/or hesperidin positively reversed the CFA-arthritis-induced increases in hind leg paw swelling, lipid peroxidation, and leukocyte count [128].

### 3.10. Role in Digestive System-Associated Pathogenesis

Hesperidin shows a role in the inhibition of digestive system-associated pathogenesis through different mechanisms (Figure 3 and Table 6).

Hesperidin showed modulatory effects on ulcerative colitis (UC) pathogenesis, which might be via lessening colonic sphingosine phosphate phosphatase 2 messenger RNA expression as well as sphingosine kinase-1 levels. Also, hesperidin improved the histopathological picture and lessened the UC disease activity index [129]. The protective role of hesperidin in DSS-induced colitis in mice and Caco-2 cells was examined. Mice with DSS-caused colitis are allocated 10, 20, and 40 mg/kg hesperidin diets after DSS treatment. In in vitro experiments, Caco-2 cells are treated with TNF-α/IFN-γ for 48 h with or without hesperidin. The supplementation of hesperidin improved colitis induced by DSS, and hesperidin ameliorates intestinal inflammation. In Caco-2 cells, it is revealed that HESP prevents TNF-α/IFN-γ-induced decrease in TEER as well as morphological disruption [50].

Hesperidin’s effect on dextran DSS-induced ulcerative colitis in mice was determined. Sulfasalazine as well as hesperidin in different doses (10, 40, and 80 mg/kg) were given orally once a day for one week, beginning alongside exposure to DSS. Oral administration of hesperidin was associated with decreased DAI, MPO activity, and MDA content. These results establish that this flavonoid can reduce colitis induced by DSS [130]. The role of hesperidin in colitis was examined. Acetic acid was induced to cause colitis in rats, and hesperidin was then given at 150 mg/kg for one week before and after colitis. It was reported that the levels of IL-6 and IFN-γ were higher in the colitis group and lower in the treatment group. Severe histopathological damage was noticed in the colitis group, whereas less damage was noticed in the treatment groups [131]. Another investigation described that pretreatment with diosmin (10 mg/kg) or hesperidin (10 and 25 mg/kg) lessened colonic injury and enhanced fluid absorption in the colon. On the other hand, hesperidin only improved colonic fluid absorption [132].

### 3.11. Role in Respiratory System-Associated Diseases

Hesperidin plays a role in inhibiting respiratory system-associated pathogenesis through multiple mechanisms (Figure 3 and Table 6). A study was conducted to examine the protective effect of hesperidin (HES) against radiation-induced lung damage in rats. The rats were exposed to a single dose of 18 Gy by cobalt-60 unit and were given HES (100 mg/kg) for one week before irradiation. The outcomes exhibited that radiation exposure caused noticeable inflammation and increased infiltration of inflammatory cells compared with the control group (G1). However, treatment with HES reduced these inflammatory responses relative to the irradiated group (G2). Furthermore, HES meaningfully alleviated pathological changes, including radiation-induced fibrosis as well as pneumonitis in lung tissue [133]. A study’s result showed that pretreatment with hesperidin protects the lungs associated with MV, potentially preventing MV-related injury [134]. How HES modulates local immune responses in the lungs during acute lung inflammation caused by LPS in vivo has been examined. The results designated that hesperidin (200 mg/kg oral administration) downregulated the expression of IL-1 beta, IL-6, MIP-2, IL-12, TNF-alpha, as well as MCP-1 induced by LPS. Additionally, the production of IL-4 and IL-10 was enhanced. Hesperidin also reduced nitric oxide production, total leukocyte counts, and iNOS expression [135].

A study was designed to examine the involvement of HMGB1 in HDN-induced immunoregulation of ALI. ALI in BALB/c mice was induced by LPS (0.5 mg/kg) intranasal administration. Hesperidin (500 mg/kg) was given intragastrically 10 days prior to exposure to LPS. Hesperidin suggestively protected animals from LPS-induced acute lung injury, as demonstrated by a reduction in histological damage in the lungs, and lower levels of macrophages, total cells, neutrophils, and MPO activity [136]. A research was designed to assess whether hesperidin (HES) was more effective than Eltroxin (ELT) in reducing lung injury induced by carbimazole (CBZ) in rats. Both HES and ELT confirmed the ability to mitigate lung injury [137], and another study reported the role of hesperidin against sepsis-induced lung injury [138]. Hesperidin demonstrated effectiveness in improving pulmonary function impaired by H1N1. It significantly declined the local numbers of immune cells as well as the concentrations of cytokines [139].

The antiasthmatic role of hesperidin was investigated. In mice, it was noticed that hesperidin-treated groups showed suppressed allergic airway inflammation, eosinophil infiltration, and airway hyperresponsiveness (AHR), and these happened through suppressing the production of OVA-specific IgE, IL-5, and IL-17 [140].

The role of hesperidin in bleomycin (BLM)-induced pulmonary fibrosis was measured. Administration of hesperidin at doses of 50 and 100 mg/kg significantly improved BLM-induced changes in lung index, oxygen saturation levels, lung function test outcomes, and the differential cell count in bronchoalveolar lavage fluid. Additionally, hesperidin treatment reduced ultrastructural and histological abnormalities [47]. A study was done to examine whether hesperidin could be effective in improving IAV-induced lung injury. A rat model of H1N1 virus infection was made and intraperitoneally given different doses of hesperidin for 5 days. Hesperidin exhibited efficiency in improving H1N1-induced impairment of pulmonary function in a dose-dependent way. Hesperidin lessened H1N1-induced impairment of pulmonary function [139]. The role of hesperidin against bleomycin (BLM)-induced pulmonary fibrosis was determined. Idiopathic pulmonary fibrosis (IPF) was induced in rats by single intratracheal BLM injection, followed by methylprednisolone or hesperidin (25, 50, and 100 mg/kg, p.o.) treatment for 4 weeks. The treatment with hesperidin restored the BLM-induced change in lung index, hematology, as well as lung function test. The changed total and differential cell count in BALF as well as blood was restored by treatment with hesperidin. BALF and lung antioxidant status were boosted by hesperidin [141].

### 3.12. Role in Skin Health

Hesperidin role in skin health evidences through different mechanisms (Table 6). The capability of hesperidin to prevent apoptosis due to oxidative stress generated by UVB-induced ROS was determined. To evaluate whether hesperidin (50 μM) itself absorbs UVB, its absorption spectrum from 200 nm to 400 nm was explored. The absorbance profile of this compound showed two protruding peaks at 227 nm as well as 283 nm. Since UVB lies between 280 and 320 nm wavelength, it approves that this compound itself can absorb UVB radiation. It is noted that hesperidin pretreatment knowingly lessened cellular macromolecule damage produced by UVB-induced oxidative stress. Pretreatment with hesperidin notably downregulated the expression of BAX and evidently upregulated Bcl-2 expression in UVB-irradiated cells [142]. Hesperidin suggestively reduced oxidative stress and inflammation caused by UVA exposure. In conclusion, treatment with hesperidin effectively protected HaCaT keratinocytes from skin damage caused by UVA radiation [143].

The therapeutic effect of hesperidin (Hes) was estimated using an imiquimod (IMQ)-induced psoriasis-like mouse model alongside lipopolysaccharide (LPS)-stimulated human immortalized keratinocyte (HaCaT) cells. In the animal model, mice were treated with IMQ and simultaneously received oral doses of Hes ranging from 125 to 500 mg per kg per day. In the in vitro experiments, HaCaT cells were exposed to LPS (1 μg/mL) and treated with Hes at concentrations of 5–20 μg/mL for 24 h, after which relevant parameters were examined. Hes has shown significant improvement in psoriasis-like skin lesions in IMQ-treated mice and has inhibited the proliferation of LPS-induced HaCaT cells. Additionally, Hes has led to a notable reduction in PASI scores, inhibited the proliferation and differentiation of epidermal cells, and decreased epidermal thickness. It has also reduced local skin lesions and serum levels of insulin and glucose as well as mRNA expression of inflammatory factors [144]. A recent study highlighted that hesperidin derived from orange peel shows promise as a beneficial bioactive ingredient for skincare [145].

A wound dressing comprising 5% hesperidin hydrogel promoted faster wound contraction and reduced the average healing time as compared with 5% naringin hydrogel or a combined hesperidin–naringin hydrogel formulation. Additionally, the hesperidin hydrogel dressing demonstrated greater collagen and DNA synthesis [146]. The role of hesperidin in UVB-induced angiogenesis in HR-1 hairless mice was investigated. The experimentation examined the role of orally given hesperidin in the dorsal skin of UVB-irradiated mice. UVB-treated mice from this compound group received oral administration of 0.1 mL of water comprising hesperidin (concentration of 100 mg/kg b.w. per day). Hesperidin treatment suggestively reduced skin neovascularization triggered through repetitive UVB light exposure. Exposure to UVB radiation leads to an increase in the expression of VEGF, as well as MMP-9 and MMP-13; however, these effects were inhibited by hesperidin treatment. It was reported that hesperidin inhibited the increase in HIF-1α expression induced by UVB exposure [147].

An experiment was conducted to assess the effects of hesperidin on PM_2.5_-induced cellular senescence, mitochondrial dysfunction, as well as cell cycle arrest in human HaCaT keratinocytes. Cells were pretreated with hesperidin (50 µM) for 30 min before exposure to PM_2.5_ (50 µg/mL). Hesperidin effectively reversed the mitochondrial depolarization caused by PM_2.5_ exposure. It also downregulated the elevated expression of phospho-p53, p21, p27, as well as p16 proteins induced by PM_2.5_, while restoring the reduced levels of cyclin E, cyclin D1, Cdk4, and Cdk2 [148].

The cytoprotective properties of hesperidin against PM_2.5_-mediated damage in a human skin cell line (HaCaT) were checked. Both hesperidin as well as NAC-treated groups exhibited lessening in PM_2.5_-induced intracellular ROS levels, and hesperidin lessened the PM_2.5_-induced intracellular ROS levels. The highest apoptotic index was found in PM_2.5_-treated cells, while the NAC- and hesperidin-treated cells exhibited a noteworthy decrease. PM_2.5_ reduced cell viability (68%), while hesperidin restored 82% cell viability. It was also noticed that PM_2.5_ raised mitochondrial depolarization, which was remarkably reversed by hesperidin. The study findings concluded that hesperidin shows therapeutic effects against PM_2.5_-induced skin damage via mitigating excessive ROS accumulation, apoptosis, and autophagy [149]. In a study involving hairless mice, topical co-treatment with glucocorticoids (GC) and 2% hesperidin effectively prevented potential damage to epidermal permeability barrier homeostasis and stratum corneum acidification typically induced by GC. This protective effect can be attributed to a significant increase in filaggrin expression, faster maturation of lamellar bilayers, and improved activity of epidermal β-glucocerebrosidase [150].

### 3.13. Role in Reproductive System-Associated Pathogenesis

The role of hesperidin in modulating the pathogenesis of reproductive system illnesses has been confirmed through multiple biological mechanisms (Figure 3 and Table 6). A study evaluated the role of hesperidin against cisplatin-caused testicular and spermatological damage in rats. Animals were divided into control and treatment groups, where cisplatin was administered as a single intraperitoneal dose of 7 mg/kg. In another group, hesperidin was administered orally at 50 mg/kg for 14 days, while a combined group received both cisplatin and hesperidin at the same dosages. The findings exhibited that hesperidin, administered at 50 mg/kg per day, employed protective effects on reproductive tissues principally by its antioxidant as well as anti-inflammatory actions. It helped reduce oxidative stress by neutralizing free radicals and strengthening the endogenous antioxidant defense system, thereby limiting cellular damage in reproductive organs [151].

The effects of hesperidin on testicular toxicity induced by BPA in rats were examined. It was noticed that the administration of hesperidin with BPA reduced oxidative stress, apoptosis, and inflammation, resulting in therapeutic effects on both spermatogenesis and steroidogenic enzymes and reproductive hormones [152]. Another study indicated that paclitaxel (2 mg/kg/bw i.p.) induced damage by elevating inflammation, endoplasmic reticulum (ER) stress, apoptosis, and oxidant levels in testicular tissue. Rats were given oral dosages of 100 as well as 200 mg/kg/bw of this compound for 10 days after paclitaxel injection. However, hesperidin demonstrated a protective effect by mitigating the decline of these levels [153]. Administration of glyphosate (GLP) led to a decline in sperm plasma membrane integrity and reduced sperm motility. It also led to increased weights of the left epididymis and the right testis, as well as higher abnormal sperm counts. Conversely, treatment with hesperidin exhibited beneficial therapeutic effects on these parameters and reduced histopathological damage caused by GLP [154].

A study was conducted to examine the role of hesperidin on colistin-induced reproductive damage in rats. Rats were divided into: control group; hesperidin group, received 300 mg/kg hesperidin daily for one week; colistin group, received 73 mg/kg colistin during 7 days; and colistin plus hesperidin group, given 300 mg/kg hesperidin daily following the colistin treatment. It was found that hesperidin supplementation reduced oxidative stress levels in the testes of the colistin plus hesperidin group compared to the colistin group. Moreover, colistin treatment increased sperm abnormality and reduced the percentage of sperm motility [155]. Mahnaz Zarein et al. 2023 reported that malathion (MAL) exposure resulted in the development of architectural and structural abnormalities of the ovaries. It was noted that HES demonstrated the ability to mitigate the harmful effects of malathion [156]. A study concluded that hesperidin exhibited protective potential against malathion-induced ovarian toxicity via modulating inflammation, cytokine production, and apoptosis [157].

### 3.14. Anti-Obesity Effects

The anti-obesity effects of hesperidin have been documented through various mechanisms (Figure 3). The effects of hesperidin on hypercholesterolemia as well as fatty liver were assessed in rats fed a cholesterol-enriched diet. Administration of hesperidin at 0.08% meaningfully reduced hepatic steatosis and liver weights, lowered adipose tissue, and reduced serum total cholesterol levels in these rats. It also diminished the significant alterations in mRNA expression of proteins associated with lipid metabolism [158].

A study examined the effects of apigenin, hesperidin, as well as kaempferol on both pre-adipocytes and mature adipocytes. In addition, the expression of genes related to triglyceride (TG) accumulation was assessed. Pre-adipocytes were cultured from day 0 to day 8, while mature adipocytes were treated for 48 h with the polyphenols at concentrations of 1, 10, and 25 µM. The results designated that apigenin, hesperidin, and kaempferol exerted anti-adipogenic and lipid-reducing (delipidating) effects in human adipocytes [159]. In a study using Caenorhaditis elegans as a model animal, it was reported that hesperidin (100 μM) reduced fat accumulation in high-fat worms and daf-2 mutant worms. Also, hesperidin (50 μM) reduced the ratio of oleic acid/stearic acid, and oleic acid supplementation restored hesperidin’s inhibitory effect on fat accumulation. Hesperidin downregulated the expression of stearoyl-CoA desaturase, fat-7, and fat-6, and fat-6 and fat-7 mutation reversed fat accumulation inhibited with hesperidin [160].

### 3.15. Role in Bone Disease

A study was performed to know the effects of hesperidin against bone loss in ovariectomized (OVX) mice. The animals were separated into several groups, together with a control diet group, including an OVX group receiving a standard diet, an OVX+HesA group fed a diet containing hesperidin (0.5 g/100 g), an OVX plus HesB group getting 0.7 g/100 g alpha-glucosyl hesperidin, and an OVX plus 17β-estradiol group administered estradiol 0.03 µg per day along with a control diet. The results exhibited that the femur was significantly reduced in OVX mice compared with the sham-operated group. However, supplementation with either hesperidin or alpha-glucosyl hesperidin effectively mitigated bone loss and helped restore bone loss [161]. The bone-sparing effect of hesperidin was evaluated in two age groups of ovariectomized rats. In older groups of rats, hesperidin intake led to partial inhibition of ovariectomy-induced bone loss, whereas younger rats showed complete inhibition [162]. The antiosteoporotic role of hesperidin against the ovariectomized (OVX) rat model of osteoporosis was examined. Hesperidin (5, 10, and 20 mg per kg) was orally given to OVX rats. Hesperidin meaningfully reduced the acid phosphatase, alkaline phosphatase, and osteocalcin levels in OVX rats. Hesperidin significantly and dose-dependently reduced the pixel density and increased bone mineral density. It significantly reduced the level of phosphorus and calcium and increased the level of vitamin D [163]. In vitro studies indicated that hesperidin partially countered the inhibition of osteogenic differentiation caused by dexamethasone by suppressing p53 activation. This suggests that hesperidin could be a promising candidate for treating osteoporosis induced by dexamethasone [164]. Hesperidin has been described to meaningfully inhibit collagen degradation, in that way increasing resistance to enzymatic breakdown as well as improving the mechanical strength of the dentin matrix [165,166].

### 3.16. Role in Eye Disease

Hesperidin’s hypotensive effect in rats with acute and chronic glaucoma, glutamate level in vitreous humor, and GSH level in aqueous humor were checked following hesperidin treatment (25, 50, and 100 mg/kg). Hesperidin treatment meaningfully lowered the elevated intraocular pressure caused by dextrose-induced ocular hypertension. Similarly, in rats with prednisolone acetate-induced ocular hypertension, hesperidin treatment markedly decreased the IOP. Hesperidin increased the glutathione levels in the aqueous humor and decreased glutamate levels in the vitreous humor. Overall, these findings suggest that hesperidin supplementation effectively helps address glaucoma [167].

A study explored the effects of hesperidin on retinal and plasma alterations in streptozotocin-induced diabetic rats. Diabetic animals were treated with hesperidin at daily doses of 100 and 200 mg/kg for a total of 12 weeks. Treatment with hesperidin was found to improve retinal thickness and prevent disruption of the blood–retinal barrier (BRB). In addition, it dropped blood glucose levels and inhibited AR activity [168]. A study was conducted to assess the effects of hesperidin in retinal damage caused by hypobaric hypoxia. Hesperidin demonstrated a protective role by promoting the activation of Nrf2 and HO-1, attenuating levels of apoptotic caspases, lowering Bax expression, and maintaining Bcl-2 levels [169]. The experimentation was conducted to examine the protective effects of hesperidin. The hesperidin intervention group was given daily intragastrical administration of hesperidin (40 mg/kg), and the hypoxia group was intragastrically administered the same dose of normal saline. It was demonstrated that hesperidin restored mitochondrial function and inhibited high glucose-mediated cell loss [170]. Hesperidin confirmed effectiveness in enhancing the antioxidant defenses, reducing ROS production triggered by high glucose levels, and preventing loss of cell viability. The findings advocate that hesperidin may offer protection to retinal pigment epithelial (RPE) cells from damage caused by high glucose by regulating the mitochondria-mediated apoptotic pathway as well as scavenging ROS [171].

### 3.17. Radioprotective Effects

Hesperidin role as radioprotective evidences through different mechanisms (Table 6). The effects of hesperidin on ovarian damage induced by ionizing radiation (IR) were examined. Hesperidin (100 mg/kg) was administered orally for 7 consecutive days. It was demonstrated that the IR group showed necrosis, atresia, and apoptosis, and that, in ovaries, increased oxidative stress and reduced estrogen and progesterone levels were observed. However, hesperidin pretreatment recovered the number of follicles and improved histological features in the ovaries of the irradiated mice. Moreover, the hesperidin decreased oxidative stress and increased estrogen and progesterone [172]. The radioprotective activity of hesperidin against genotoxicity caused by gamma-irradiation was investigated. For each volunteer, the outcomes exhibited a significant increase in the incidence of micronuclei after exposure of cells to gamma-irradiation. The lymphocytes in the blood samples collected at 1 h after hesperidin ingestion and exposed in vitro to gamma rays exhibited an imperative decrease in the incidence of micronuclei, as compared with the same irradiated lymphocytes from blood samples collected at 0 h. The best protection and reduction in frequency of micronuclei (33%) was noticed at 1 h after hesperidin ingestion [173]. Pre-administration of an effective dose of hesperidin [25 mg/kg body weight] altered the radiation-induced effects by reducing lipid peroxidation levels and DNA damage. It improved the antioxidant status, bringing it close to normal, while decreasing both the lipid peroxidative index and comet parameters. Moreover, hesperidin treatment caused a reduction in the hepatic injury induced by radiation [174]. Research findings reported that treatment with hesperidin before or after exposure to γ-radiation decreased oxidative stress, apoptosis, and LPO [175]. A study was performed to assess the radioprotective potential of hesperidin in an experimental rat model. The rats were exposed to a dose of 18 Gy of 6 MV X-ray. Then, 100 mg per kilogram doses of HES were administered for one week before irradiation. It was reported that hesperidin administration decreases fibrosis, inflammation, mast cell numbers, macrophage, and myocyte necrosis [176].

The efficiency of HES against the severity of biochemical ailments in the cerebral hemispheres of irradiated rats was investigated. HES (50 mg/kg b.w) was given for 10 successive days before exposing the whole body to gamma rays (5 Gy) and during 2 weeks after irradiation. Hesperidin treatment meaningfully reduced monoamine alterations, oxidative stress, as well as mitochondrial damage in the cerebral hemispheres of irradiated rats [177].

### 3.18. Role in Oral Health

A study investigated the impact of hesperidin on the demineralization of human root dentin and the preservation of collagen, comparing its effects with those of grape seed extract and chlorhexidine. Both the grape seed extract and hesperidin groups showed a reduction in demineralization while maintaining the integrity of the collagen matrix. Further, the hesperidin group exhibited the lowest values for lesion depth and mineral loss [178]. A study examined the antibacterial and cytotoxicity effects of a total etch dentin adhesive that was incorporated with hesperidin. It was reported that dimethyl sulfoxide, total etch bonding agent, and hesperidin work together synergistically to combat E. faecalis, L. acidophilus, and S. mutans [179]. A study explored the beneficial effects of hesperidin in rats with ligation (Lig)-induced periodontitis. HES (75 or 150 mg/kg) was given instantly after ligature placement by intragastric feeding. The findings from dental micro-CT as well as radiographic examinations showed a substantial increase in alveolar bone loss in the Lig group, which was likely mitigated by HES treatment. The immunostaining outcomes exhibited that the ligation-induced iNOS expression was also reduced by HES. Oral administration of HES has a beneficial effect on preventing bone loss [180].

### 3.19. Immunomodulatory Effects

Hesperidin reveals notable immunomodulatory properties in a variety of studies. In a study in which a tumor model was made, cisplatin plus hesperidin synergism and immunomodulatory effects were assessed. The findings designated that hesperidin stimulated anti-tumor immunity by increasing the proportion of T helper (CD3+CD4+) and T cytotoxic (CD3+CD8+) cells [181]. A study was conducted to assess the role of hesperidin in the regulation of the immune response in experimental animal models. All tested doses of hesperidin caused a notable immunomodulatory response in a sheep red blood cell challenge model [182]. A study intended to evaluate both the in vitro as well as in vivo bactericidal and immunomodulatory roles of hesperidin and ellagic acid against *Aeromonas hydrophila*. Treatment with hesperidin as well as ellagic acid proved antimicrobial activity against *A. hydrophila*. Both compounds also meaningfully raised anti-LPS IgM levels, while declining the elevated anti-LPS and anti-ECP IgA levels back toward normal [183].

**Table 6:** Role of hesperidin in the management of various pathogeneses by mechanisms such as anti-inflammatory, antioxidant, and preserving different organ/tissue structures.

Activity	Study Model	Doses	Findings	Ref.
Anti-cancer effects	Lung cancer mice model	100 mg/kg	° Reduced tumor volume and tumor weight	[120]
Anti-arthritis effects	Collagen-induced arthritis rat model	160 mg/kg	° Inhibits arthritis via reduction in neutrophil activation	[126]
	Collagen-induced rheumatoid arthritis rat model	50 mg/kg	° Improve severity of rheumatoid arthritis	[127]
	Freund’s adjuvant-induced arthritis rat model	25 mg/kg	° Hesperidin administration caused ameliorative effects on arthritis	[128]
Anti-colitis effects	Dextran sulphate sodium -induced colitis mice model	10,40, 80 mg/kg	° Decreased MPO activity, MDA and IL-6 level	[130]
	Trinitrobenzenesulfonic acid colitis rat model	10, 25 mg/kg	° Hesperidin prevents colonic inflammation and improved colonic fluid absorption	[132]
Lung protective effects	Lipopolysaccharide-induced acute lung injury mice model	500 mg/kg	° Protects mice from LPS induced acute lung injury	[136]
	Sepsis-induced lung injury mice model	10 and 20 mg/kg	° Histopathological changes reduced	[138]
Skin injury protection potential	UV radiation-induced skin damage in HaCaT cells	220 μg/mL	° Hesperidin treatment protected keratinocytes	[143]
Protective effects on reproductive system toxicity	Cisplatin-induced testicular and spermatological damages rat model	50 mg/kg	° Protects testicular and stemmatological damages	[151]
	Bisphenol-induced testicular toxicity rat model	50 mg/kg	° Alleviates inflammation in testicles ° It has regulatory effects on serum reproductive hormones, spermatogenesis	[152]
	Paclitaxel-induced testicular toxicity rat model	100 and 200 mg/kg	° Protective effect on testicular toxicity	[153]
Bone-sparing effects	Ovariectomized-induced osteopenia rat model	Standard diet supplemented with 0.5% hesperidin	° Hesperidin inhibits osteopenia	[162]
Protective effects on retinal abnormalities	Retinal abnormalities in diabetic rat model	100, 200 mg/kg	° Attenuates retina anomalies	[168]
Radioprotective effect	X-ray radiation-induced cellular damage of liver mice model	12.5, 25, 50 and 100 mg/kg	° Declined the hepatic damage	[174]
Effect on periodontitis	Ligation-induced periodontitis rat model	75 or 150 mg/kg	° Hesperidin showed ameliorative effect against the alveolar bone loss	[180]
Immunomodulatory Effects	Ehrlich Ascites Carcinoma Tumor mice model	100, 200 mg/kg	° It stimulated anti-tumor immunity	[181]

### 3.20. Antimicrobial Activity

Hesperidin exhibits significant antimicrobial properties, making it a promising candidate against various pathogens. Its antimicrobial action is mainly due to its capacity to disrupt pathogen cell membranes, inhibit metabolic processes, and interfere with biofilm formation. A study investigated the potential of hesperidin to inhibit biofilm formation and reduce MRSA virulence. The results indicated that hesperidin treatment notably inhibits the production of lipase, autoaggregation, hemolysin, autolysin, and staphyloxanthin. The decrease in staphyloxanthin production may enhance the susceptibility of MRSA to H_2_O_2_ oxidative stress conditions. The findings on gene expression revealed that hesperidin treatment led to the downregulation of several key factors, including the biofilm-associated gene, the polysaccharide intracellular adhesion gene, fibronectin-binding protein, autolysin, and the production of staphyloxanthin [184]. Hesperidin significantly inhibited the growth and proliferation of parasites and effectively reduced the growth of intra-macrophagic amastigotes [185]. A study’s finding designates that hesperidin microemulsion showed antibacterial activity against various bacteria [186]. A study was conducted to study whether hesperidin could be effective in improving influenza A virus (IAV)-induced lung injury. It was reported that hesperidin presented efficacy in the improvement of H1N1-induced impairment of pulmonary function in a dose-dependent manner [139]. A study was conducted to measure the role of hesperidin against ovalbumin (OVA)-induced bronchial asthma. It was noticed that hesperidin reduced alveolar sac damage, cellular infiltration, bronchiole wall disruption, as well as nuclei pyknosis in neuron cells. Lastly, hesperidin may deliver safety against OVA-induced asthma [187].

A study aimed to appraise both the in vitro and in vivo bactericidal and immunomodulatory effects of hesperidin and ellagic acid against *Aeromonas hydrophila*. Treatment with hesperidin (HES) as well as ellagic acid (EA) proved antimicrobial activity against *A. hydrophila*. Both compounds also meaningfully raised anti-LPS IgM levels, while declining the elevated anti-LPS and anti-ECP IgA levels back toward normal, as compared to the infected group, which showed decidedly increased levels two weeks after infection [183].

## 4. The Synergistic Effects of Hesperidin When Combined with Other Drugs or Compounds

**Figure 4:**
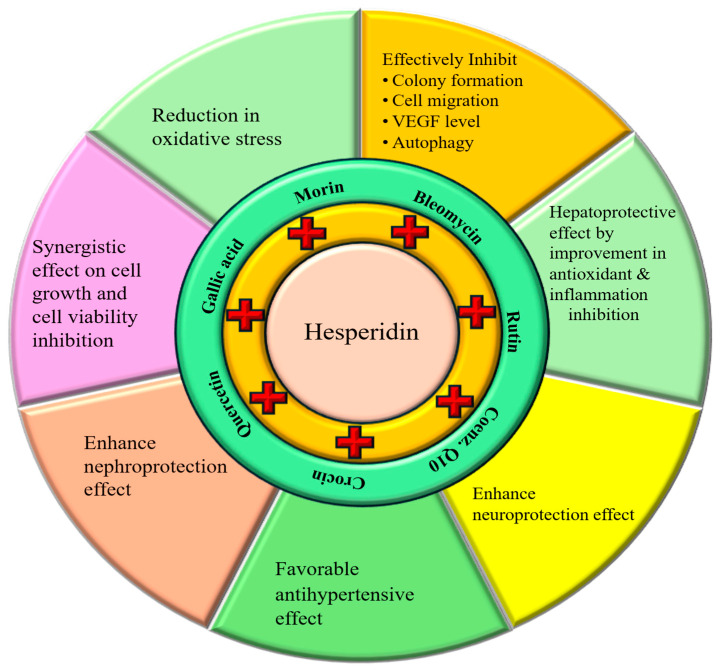
Synergistic effects of hesperidin with various drugs and bioactive compounds. Several studies designate that hesperidin exhibits synergistic interactions with compounds/drugs, including morin, coenzyme Q10, bleomycin, rutin, crocin, quercetin, and gallic acid. These interactions improve diverse biological properties such as inhibition of cancer cell proliferation, hepatoprotective activity via antioxidant and anti-inflammatory pathways, neuroprotection, nephroprotection, and antihypertensive effects. Moreover, combined treatments often demonstrate improved efficacy in suppressing cell growth and viability. The information presented in this figure is compiled from references [87,190,191,192,193,194,195].

Several studies have reported that hesperidin shows synergistic effects with other drugs or compounds, acting by different mechanisms across a range of pathogeneses (Figure 4 and Table 7). A study was conducted to examine whether DOX efficiency is improved by hesperidin (Hsd) in metastatic breast cancer, 4T1. Hsd synergistically improved the cytotoxic effect of DOX, which appeared to relate to an increase in apoptotic cell death, G2/M cell cycle arrest, as well as blocked migration of 4T1 cells [188].

A study finding concluded that bee venom, hesperidin, and piperine work synergistically to improve the efficacy of tamoxifen in managing breast cancer [189]. Another study concluded that hesperidin, as well as gallic acid, employs synergistic potential on cell growth, spheroids, and stemness of colorectal cancer [190].

**Table 7:** The synergistic or combined effects of hesperidin with other drugs or compounds.

Hesperidin	**Drugs/Compounds**	**Outcome**	**Ref.**
	Tamoxifen	° Bee venom, hesperidin, and piperine work together to boost and enhance the effectiveness of tamoxifen in treating breast cancer.	[189]
	Gallic acid	° Gallic acid as well as hesperidin work together synergistically to influence cell growth and spheroid formation in colorectal cancer	[190]
	Bleomycin	° Hesperidin + bleomycin combination amplified antiproliferative as well as anti-angiogenic effects.	[192]
	Rutin	° The cotreatment of rutin as well as hesperidin is effective in restoring the histological integrity and liver function	[87]
	Coenzyme Q10	° Hesperidin in combination with coenzyme Q10 improves mitochondrial functions and reduce psychotic symptoms	[193]
	Crocin	° Co-administration of hesperidin and crocin exhibited antihypertensive effects	[194]
	Quercetin	° A combination of quercetin and hesperidin showed free radical scavengers and reduced nephrotoxicity	[195]

A study was conducted to determine the synergistic role of morin and hesperidin against oxidative stress in hepatotoxicity. It was demonstrated that cisplatin administration raised nitric oxide and malondialdehyde levels and also reduced the catalase and glutathione levels in the liver. However, in the morin and/or hesperidin groups, myeloperoxidase activity, nitric oxide and malondialdehyde levels, glutathione level, and catalase activity were higher than in the cisplatin-induced group [191]. A study based on lung cancer cells exhibited that the hesperidin + bleomycin combination meaningfully amplified anti-angiogenic, antiproliferative, and autophagic apoptotic activities in comparison to any treatment alone. The therapy with a combination efficiently inhibited cell migration as well as colony formation, whereas evidently, VEGF levels were reduced, demonstrating anti-angiogenic properties [192]. A study was designed to determine if hesperidin, rutin, and their combination protect against hepatotoxicity. It was reported that the treatment of paclitaxel administered with hesperidin as well as rutin meaningfully diminished paclitaxel-induced increases in AST, ALT, gamma-glutamyl transferase, ALP, as well as liver lipid peroxidation and total bilirubin level [87]. The protective role of hesperidin alone or in combination with coenzyme Q10 against ketamine-induced psychotic symptoms in mice was determined. The study exhibited that hesperidin alone or in combination with coenzyme Q10 improves mitochondrial functions and antioxidant systems and reduces psychotic symptoms in mice, suggesting a neuroprotective role against psychosis [193].

The role of hesperidin (HES) and crocin (CRO), administered alone as well as in combination, in blood pressure was estimated in a hypertensive rat model. After 49 days of a high-fat diet (HFD), the rats exhibited a noticeable increase in heart rate, systolic blood pressure, and mean arterial pressure when compared to the control group. In contrast, rats getting the combined treatment of CRO and HES alongside the HFD revealed reduced heart rate and blood pressure parameters. Overall, the co-administration of hesperidin and crocin confirmed noteworthy antihypertensive potential [194]. Another study’s result reported that the combination of hesperidin and quercetin acts as a free radical scavenger and antioxidant together, and they might be capable of decreasing nephrotoxicity caused by carbon tetrachloride [195].

## 5. Role of Hesperidin-Based Nanoformulation in Management of Pathogenesis

Hesperidin plays a useful role in managing various pathogeneses; however, its therapeutic potential is limited by its poor bioavailability. To address this issue, numerous studies have focused on enhancing its bioavailability through different types of nanoformulation. These nanoformulations aim to tackle the challenges of low bioavailability and toxicity, thereby improving the efficacy of hesperidin in treating various pathogenesis compared to the native compound. Several types of nanoformulations have been synthesized, and their efficacies have been evaluated through both in vitro and in vivo studies (Table 8). Below, we discuss some hesperidin-based nanoformulations.

Gold nanoparticles loaded with hesperidin (Hsp-AuNPs) were synthesized, and their cytotoxic effects were examined in the human breast cancer cell line MDA-MB-231. The synthesized Hsp-AuNPs confirmed greater anti-cancer effects against MDA-MB-231 breast cancer cells when compared to hesperidin or AuNPs alone, revealing dose-dependent cytotoxicity. Among the tested agents, the treatment with Hsp-AuNPs caused higher functional activities of macrophages in Ehrlich ascites tumor cell-bearing mice when compared to the control group. The production of IL-1β, IL-6, and TNF-α was measured in bone marrow derivative macrophage cells exposed to Hsp-AuNPs at 50 or 250 µg mL^−1^. The outcomes deliver a clear sign that Hsp-AuNPs meaningfully reduced the production of all of the above-mentioned cytokines, when compared to that of LPS induction, in a concentration-dependent way [196]. Ion gelation was used to make a chitosan/hesperidin nanoformulation. Male albino mice received a pretreatment of 100 mg/kg of Hes or Hes-NPs, which was given daily for two weeks before the induction of doxorubicin nephrotoxicity on the 12th day. It was reported that doxorubicin’s nephrotoxic effects were reduced by pretreatment with Hes or Hes-NPs, with Hes-NPs reporting the greatest reduction [197]. Hesperidin-loaded N-carbon nanoparticles (N-CNPs) were synthesized, and their bactericidal potential was studied. Gram-negative *Escherichia coli* (*E. coli*)-sensitive (ATCC 10,536) as well as resistant (ATCC 35,218) strains were utilized for the antibacterial effects. An increased bactericidal activity of hesperidin was noticed against the tested bacterial strains. The results indicate the potential of N-carbon nanoparticles as an effective vehicle for delivering hesperidin, thereby improving bactericidal activity [198]. A study examined the view of developing a carrier system for the effective delivery of hesperidin by selectively targeting HSCs in a fibrotic rat model. The liposomes were investigated for effective targeting of the hepatic stellate cells in the treatment of liver fibrosis. The bio-distribution data of the formulated carrier system designates more uptake of hesperidin principally by the fibrotic liver with an insignificant amount in non-targeted organs, which is surely valuable due to lower systemic distribution and low risk of toxicity. The developed carrier signifies a potentially useful approach for HSCs’ specific targeting of hesperidin in a liver fibrosis rat model [199]. The high effectiveness of alginate as well as chitosan hydrogels in wound healing was assessed by loading numerous concentrations of hesperidin into these hydrogels, followed by an assessment of their antibacterial activity and toxicity properties. The therapeutic role of the prepared hydrogels was assessed in the full-thickness dermal wound in a rat model. The results designated that the hydrogels have suitable porosity (91.2 ± 5.33%) with interconnected pores. Furthermore, the time-kill assay exhibited the antibacterial effects of hydrogels, and the MTT assay demonstrated the positive effect of hydrogels on cell proliferation and did not show toxicity activities on cells. Also, the in vivo outcomes designated that the made hydrogels caused better wound closure than the gauze-treated wound, and the maximum wound closure % was noticed for the alginate/chitosan/10% hesperidin group [200].

A dendrimer-based hydrogel bandage was formulated to enhance the healing of full-thickness wounds, and its therapeutic efficacy was assessed using an animal model. Wound progression was checked from day 0 to day 14 across different groups, including a control group, hesperidin gel, and Hsp-PAMAM formulations at concentrations of 2.5%, 5%, 7.5%, and 10%. All groups confirmed gradual wound contraction over time; however, the hesperidin gel showed relatively faster healing. In contrast, the control group exhibited signs of infection and inflammation, along with incomplete wound closure. Among the tested formulations, Hsp-P-Hyd 10% achieved the most rapid wound healing, with complete closure and no evidence of infection or inflammation. Histopathological analysis of the hesperidin gel group revealed the formation of a new epidermal layer at the wound site, with the surface fully covered by granulation tissue and an epithelium layer. Notably, the Hsp-P-Hyd 10% formulation resulted in the greatest wound contraction, formation of epidermal layer, and effective tissue remodeling [201].

A further investigation was conducted to develop a phospholipid complex of hesperidin aimed at enhancing its dissolution and, consequently, its oral bioavailability. The partition coefficient and solubility of the resulting complexes confirmed substantial improvement compared to the free drug. Precisely, free hesperidin displayed a drug release of 46.9%, while the hesperidin–phospholipid complex (F2) achieved a release of 78.20%. The optimized formulation revealed concentration-dependent antioxidant action [202]. Hesperidin was incorporated into nanoemulsions (HP-NEM) using a spontaneous emulsification method to improve its solubility, bioavailability, and effectiveness in breast cancer treatment. Cytotoxicity assessments performed with the MTT assay demonstrated that this formulation selectively targeted MCF-7 cells. The treatment with HP-NEM triggered cell death through apoptosis and caused cell cycle arrest in the G2/M phase in MCF-7 cells. This study suggests that this formulation could serve as a promising therapeutic agent for breast cancer treatment [203]. A newly developed formulation of HESP-lowered solid lipid nanoparticles (SLNs) was made to enhance solubility and bioavailability. Electron microscopy analysis revealed that the nanoparticles have a spherical shape. The optimization process yielded HESP-SLNs with a small size of 179.8 ± 3.6 nm and demonstrated high encapsulation efficiency. The anti-inflammatory activity of HESP-SLNs was determined in xylene-induced ear edema in a mouse model. HESP-SLN formulation caused a better anti-inflammatory effect than either HESP cream or HESP-free SLNs. The ear thick connective tissue of the mouse treated with HESP-SLNs demonstrated enhanced anti-inflammation effect as compared to HESP cream [204]. HES-loaded solid lipid nanoparticles (SLNs) were synthesized to enhance the oral delivery of HES. Pharmacokinetic studies in rats indicated that HES from the SLN formulation had approximately 4.5 times higher bioavailability than HES in suspension. The cardioprotective activity of HES-SLNs was assessed in the DOX-induced cardiotoxicity in the rat model. HES-SLN effectively reduced DOX-induced cardiotoxicity and improved histopathological scores as compared to the DOX group. Also, treatment with HES-SLNs efficiently reduces DOX-induced cardiotoxicity through oxidative stress and apoptosis suppression [205]. Hesperidin (HPN) incorporated into polyacrylonitrile (PAN)/polyethylene oxide (PEO) electrospun nanofibers was developed for use as wound dressings. In vivo experiments showed that the wound healing rate for the HPN-loaded nanofibers was better compared to other groups. Additionally, histological assessments using H&E and MT staining revealed that the HPN-loaded nanofibers significantly enhanced skin regeneration compared to HPN-free nanofibers [206].

Hes-PLGA-NPs were developed, and in vitro studies conducted on HEp-2 cells suggest that these formulated nanoparticles are more effective than native hesperidin. This designates their potential as a strong candidate for anti-cancer treatment [207]. A study was carried out to assess how effective the co-delivery of clarithromycin (CLR) and hesperidin (Hesp) using nanostructured lipid carriers (NLCs) is against H. pylori. The NLCs were delivering sustained and controlled drug release, potentially enhancing the eradication rates of H. pylori [208]. A different examination was carried out to reconnoiter the drug delivery and effectiveness of antioxidants in the HCT116 colorectal cancer cell line through the incorporation of hesperidin into PLGA nanoparticles. The results revealed that the most significant reduction in cell viability occurred at a concentration of 10 µg/mL of PLGA nanoparticles containing hesperidin. These outcomes indicate that PLGA nanoparticles loaded with hesperidin play a vital role in significantly reducing the survival rate of colorectal cancer cells [209]. Chitosan/hesperidin nanoparticles (Hes-Nanoparticles) were synthesized. The outcomes demonstrated that Hes-Nanoparticles have better antioxidant effects than chitosan and hesperidin only. Also, it has been confirmed that formulations are more effective in early cell cycle arrest, and increase cell apoptosis and decrease the viability of cancer cells than chitosan and hesperidin alone [210].

**Table 8:** Role of formulated hesperidin in the management of pathogenesis such as enhancing the anti-inflammatory and anti-cancer activities, reduction of liver function enzymes, maintaining liver tissue structure, and reduction of nephrotoxicity and cardiotoxicity.

Formulation	Study Types	Study Model/Cell Line	Activity	Findings	Ref.
Hesperidin loaded on gold nanoparticles	In vitro	Human breast cancer cell line (MDA-MB-231)	Anti-cancer potential	° Hsp-AuNPs hold strong cytotoxicity as well as enhancement of apoptosis in cancer cells	[196]
Chitosan/hesperidin nanoformulation	In vivo	Doxorubicin-induced nephrotoxicity mice model	Nephrotoxicity protective effect	° The formulation treatment reduces renal toxicity by lessening oxidative stress and inflammation	[197]
Alginate/chitosan hydrogel containing hesperidin	In vivo	Full-thickness dermal wound in a rat model	Wound healing effect	° Prepared hydrogels caused wound better closure	[200]
Hesperidin-loaded dendrimer-based hydrogel bandages	In vivo	Full-thickness wound in a rat model	Wound healing effect	° Preparation had better wound contraction activity	[201]
Hesperidin-loaded nanoemulsions	In vitro	Breast cancer cell lines (MCF-7)	Anti-cancer effect	° Treatment with the formulation induced cell death through apoptosis, cell cycle arrest in the G2/M phase, and downregulated miR-155 and miR-21 expression	[203]
Hesperidin-loaded solid lipid nanoparticles	In vivo	Ear edema in mouse model	Anti-inflammatory effect	° This formulation improves the anti-inflammatory potential of hesperidin	[204]
HES-loaded solid lipid nanoparticles	In vivo	DOX-induced cardiotoxicity rat model	Cardioprotective effects	° The cardioprotective effect for nanoformulation against cardiotoxicity facilitated via suppression of oxidative stress as well as apoptosis	[205]
Hesperidin-loaded polyacrylonitrile/polyethylene oxide nanofibers	In vivo	Full thickness excisional wound rat model	Wound dressings	° Wound healing rate of formulation was higher with the treatment with formulation	[206]
Hesperidin-loaded PLGA nanoparticles	In vitro	Human laryngeal squamous cell carcinoma cell line (HEp-2)	Anti-cancer effect	° This nanoformulation is more capable as a potent anti-cancer candidate.	[207]
Hesperidin-loaded PLGA nanoparticles	In vitro	Colorectal cancer cell line (HCT116)	Anti-cancer effect	° The formulated nanoparticles reduce the survival rate of cancer cells	[209]
Chitosan/Hesperidin Nanoparticles	In vitro	Breast cancer cell line (MDA-MB-231)	Anti-tumor effect	° Cells treated with Hes-Nanoparticles showed a sharp G2/M-phase cell-cycle arrest ° Cells treated with Nano-Hes exhibited an increase in apoptotic cells	[210]

## 6. Bioavailability and Clinical Trials

The practical limitations of utilizing flavonoids in medical, pharmaceutical, food, and cosmetic applications are primarily determined as their chemical and biophysical characteristics, which include low solubility, chemical stability, bioavailability, and pharmacokinetics influenced by metabolic stability in the liver, intestines, and gut microflora, and challenges in plant production, such as the very low yield of these secondary metabolites compared to plant biomass, as well as issues related to enhancing biosynthesis and the complexities involved in isolation, extraction, and purification processes [211].

The chemical structure of flavonoids consists of an organic skeleton with a C6–C3–C6 aromatic ring, and the bioavailability of flavonoids is usually very low [212,213]. Hesperidin’s therapeutic potential is greatly limited by its low oral bioavailability, which is a result of its poor water solubility and restricted permeability through the intestinal epithelium.

The low bioavailability of hesperidin is largely attributed to its sugar component, the rutinose disaccharide, which is attached to the hesperetin molecule. This structure impedes absorption in the small intestine [214,215]. Consequently, the majority of ingested hesperidin needs to be metabolized in the colon by α-rhamnosidase and β-glucosidase enzymes, which convert it into hesperetin, the aglycone form [216].

The low solubility of these compounds results in only small amounts of free molecules being released into the aqueous environment of the gastrointestinal tract, which in turn limits their ability to permeate biological membranes [217]. In addition, hesperidin and hesperetin encounter another barrier to absorption in the gastrointestinal tract: both serve as substrates for P-glycoprotein [218]. Several studies reported that hesperidin shows low water solubility and restricted bioavailability [219,220,221].

Although hesperidin has been broadly inspected in in vitro and animal studies and proves noteworthy antioxidant, anti-inflammatory, and cardioprotective properties, evidence from human clinical trials is still scarce. A clinical study was performed to assess its efficacy in improving metabolic disturbances in individuals with metabolic syndrome (MetS). In this clinical trial with a parallel-group design, 49 participants with MetS were administered either 500 mg of hesperidin or a placebo twice daily for 12 weeks. Compared with the placebo group, hesperidin supplementation resulted in decreases in fasting blood glucose, TNF-α, triglycerides, and systolic blood pressure. Within-group analysis further revealed important declines in serum levels of glucose, insulin, triglycerides, low-density lipoprotein cholesterol, total cholesterol, TNF-α, as well as hs-CRP in the hesperidin-treated group, whereas the control group showed reductions only in glucose and insulin levels. These findings suggest that hesperidin supplementation may efficiently improve metabolic parameters and decrease inflammation in patients with MetS [222]. A study examined the mechanisms underlying the role of hesperidin intake in individuals with elevated blood pressure or stage 1 hypertension by analyzing changes in their transcriptomic profiles following both a single dose and a 12-week intervention. Consumption of a single dose of enriched orange juice (EOJ) was found to offer protection against insulin resistance. Furthermore, long-term intake over 12 weeks led to a decrease in the expression of pro-inflammatory genes, providing a possible mechanism of action on the inflammation pathway [223]. Furthermore, little is acknowledged about the clinical characteristics of this compound, for example, the bioavailability, the appropriate dose, tolerance, and effectiveness of hesperidin and its metabolites for human disease [224].

A study was conducted to evaluate sustained and acute effects, as well as the effect of continued consumption of orange juice (OJ) containing hesperidin as well as hesperidin-enriched orange juice (EOJ) on blood pressure (BP) and pulse pressure (PP) in individuals with pre-hypertension and stage-1 hypertension. It was noticed that a single EOJ dose (500 mL) led to reductions in systolic blood pressure (SBP) and PP, with more pronounced improvements observed after prolonged intake, including a decrease in diastolic blood pressure (DBP). Over the 12-week period, SBP and PP diminished in a dose-dependent way relative to the hesperidin content of the beverages. Both EOJ and OJ also lowered homocysteine levels compared to the control beverage. After 12 weeks of EOJ consumption, four genes associated with hypertension were differentially expressed in peripheral blood mononuclear cells. These findings advocate that EOJ may serve as a useful approach for managing BP and PP in individuals with pre- or stage-1 hypertension [225].

## 7. The Safety Level of Hesperidin

Current evidence indicates that hesperidin is generally safe and well-tolerated when consumed at typical supplemental or dietary levels, referring to intake amounts generally obtained from diet or standard supplementation. A study by Matsumoto et al. (2019) evaluated glucosyl hesperidin in rats for teratogenicity, genotoxicity, as well as acute and sub-chronic toxicity. A dose of 5000 µg/mL showed no evidence of genotoxicity and resulted in zero mortality. These findings further support the favorable safety profile of hesperidin [226].

A study based on female rats in a sub-chronic toxicity study reported that hesperidin (250 and 500 mg/kg) did not induce any abnormalities in body weight, food consumption, clinical signs, neurological and ophthalmological observations, urine analysis, and blood parameters. Hesperidin has a median lethal dose (LD_50_) of 4837.5 mg/kg and a Low Observed Adverse Effect Level (LOAEL) at 1000 mg/kg. Therefore, hesperidin isolated from citrus fruit showed a good safety profile in the animal study [227]. A single oral dose of the naringenin–hesperidin molar combination (MIX–160) at 50, 300, and 2000 mg/kg did not produce any signs of acute toxicity in rats. These findings advocate that this combination is well-tolerated, as it caused no mortality or obvious clinical side effects when administered daily at a dose of 161 mg/kg over a 28-day period [228].

## 8. Conclusions and Future Prospectives

Fruits, vegetables, as well as beverages are good sources of flavonoids, which contribute meaningfully to human health due to their potent anti-inflammatory and antioxidant properties. These bioactive compounds help neutralize free radicals, decrease oxidative stress, and modulate key signaling pathways involved in inflammation. Among these, hesperidin, a main flavonoid mainly found in citrus fruits such as oranges and lemons, has attracted substantial attention for its therapeutic role.

Hesperidin has confirmed a wide range of pharmacological potential, including antioxidant, anti-inflammatory, anti-apoptotic, and cardioprotective properties. Its ability to regulate inflammatory mediators and improve cellular antioxidant defense systems makes it mostly valued in the prevention and management of chronic diseases. Remarkably, hesperidin has shown promising effects in combating various health issues such as diabetes mellitus, liver and lung diseases, reproductive system disorders, arthritis, and cancer. In addition, its role in eye diseases, oral health management, digestive system-associated diseases, and cardiovascular and neuro-related pathological conditions has been reported in both in vivo and in vitro studies. Hesperidin enhances the effectiveness of other drugs and improves their potential for disease prevention. While it plays an important role in managing various pathogeneses, its limited bioavailability constrains its therapeutic benefits. To address this issue, many studies have focused on enhancing its bioavailability through advanced nanoformulation strategies to overcome challenges associated with low absorption and potential toxicity. Clinical trial-based studies should be conducted to further investigate the safety, efficacy, toxicity, mechanisms of action, and therapeutic implications of hesperidin for disease treatment and prevention.

## Figures and Tables

**Table 5:** Neuroprotective property of hesperidin through the reduction of memory impairment and attenuation of neurotoxicity.

Study Model	Doses	Animal Species	Treatment Duration	Key Findings	Ref.
Methotrexate-induced changes in neurogenesis rat model	100 mg/kg	Sprague–Dawley rats	21 days	° Hesperidin prevents neurotoxic effects via enhancing hippocampal neurogenesis	[107]
Fluoride-induced neurobehavioral changes rat model	200 mg/kg	Sprague–Dawley rats	8 weeks	° Neurobehavioral impairment reduced	[108]
Mild traumatic brain injury mice model	50 mg/kg	Male NMRI mice	14 days	° Decreased oxidative damage and neuroinflammation	[109]
6-Hydroxydopamine-induced Parkinson disease rat model	50 mg/kg	Female C57 BL/6 mice	28 days	° Prevented memory impairment	[110]
Aluminum chloride-induced Alzheimer disease rat model	100 mg/kg	Male Wistar rats	60 days	° Prevented biochemical anomalies, cognitive deficits	[114]
NO-mediated cerebral ischemic reperfusion injury rat model	50, 100 mg/kg	Male Wistar rats	7 days	° Improved neurobehavioral alterations, and mitochondrial complex enzyme activities	[115]
Scopolamine-induced amnesia in mice model	100, 200 mg/kg	Adult Swiss albino mice	10 days	° Co-treatment with hesperidin reversed biochemical and memory alterations	[116]

## Data Availability

No new data were created or analyzed in this study. Data sharing is not applicable to this article.
